# A reasonable approach for the generation of hollow icosahedral kernels in metal nanoclusters

**DOI:** 10.1038/s41467-021-26528-w

**Published:** 2021-10-26

**Authors:** Xi Kang, Xiao Wei, Xiaokang Liu, Sicong Wang, Tao Yao, Shuxin Wang, Manzhou Zhu

**Affiliations:** 1 Department of Chemistry and Centre for Atomic Engineering of Advanced Materials, Anhui Province Key Laboratory of Chemistry for Inorganic/Organic Hybrid Functionalized Materials, Anhui University, Hefei, Anhui People’s Republic of China; 2 Key Laboratory of Structure and Functional Regulation of Hybrid Materials, Anhui University, Hefei, Anhui People’s Republic of China; 3 National Synchrotron Radiation Laboratory, University of Science and Technology of China, Hefei, Anhui People’s Republic of China

**Keywords:** Structural properties, Synthesis and processing, Organic-inorganic nanostructures

## Abstract

Although the hollow icosahedral M_12_ kernel has been extensively observed in metal nanoclusters, its origin remains a mystery. Here we report a reasonable avenue for the generation of the hollow icosahedron: the kernel collapse from several small nano-building blocks to an integrated hollow icosahedron. On the basis of the Au alloying processes from Ag_28_Cu_12_(SR)_24_ to the template-maintained Au_x_Ag_28-x_Cu_12_(SR)_24_ and then to the template-transformed Au_12_Cu_y_Ag_32-y_(SR)_30_, the kernel evolution/collapse from “tetrahedral Ag_4_ + 4^∗^Ag_3_” to “tetrahedral Au_4_ + 4^∗^M_3_ (M = Au/Ag)” and then to “hollow icosahedral Au_12_” is mapped out. Significantly, the “kernel collapse” from small-sized nano-building blocks to large-sized nanostructures not only unveils the formation of hollow icosahedral M_12_ in this work, but also might be a very common approach in constructing metallic kernels of nanoclusters and nanoparticles (not limited to the M_12_ structure).

## Abstract (web summary)

The origin of the hollow icosahedral M_12_ kernel in metal nanoclusters is under debate. Here the authors demonstrate the Au alloying-induced kernel collapse from small-sized nano-building blocks as a viable approach for the generation of hollow icosahedral M_12_ kernel in metal nanoclusters.

## Introduction

Metal nanoclusters are an emerging class of modular nanomaterials^1–6^, and have been sparking great research interests owing to their atomically precise structures and intriguing properties^7–27^. The physiochemical properties of these nanomaterials, such as chirality, luminescence, catalysis, magnetism, and electrochemistry, can be rationalized in terms of their quantum size effect as well as discrete electronic states^28–38^. Besides, the atomically precise nature of these modular nanomaterials is of the most interest — indeed, compared with large-sized nanoparticles, nanoclusters (typically <2 nm of the metallic kernel) present more precise compositions/constructions, and thus allow for the atomic-level elucidation of structural evolutions and structure-property correlations^1–6,39–46^.

Of all reported nanoclusters with precise structures, the icosahedral configuration is the most typical, which is frequently observed in both metal kernels and ligand shells of nanoclusters^47–51^. Interestingly, except for the non-hollow icosahedral M_1_@M_12_ kernel (M represents the metal), the hollow icosahedral M_12_ kernel has also served as a basic nano-building block of nanoclusters (e.g., Ag_44_(SR)_30_, Au_12+*n*_Cu_32_(SR)_30+*n*_, Ag_50_(dppm)_6_(SR)_30_, Au_144_(SR)_60_, etc.)^10,11,52–56^. Structurally, it is accepted that the non-hollow icosahedron might be more energetically favorable than the corresponding hollow one due to the extra 12 metal···metal interactions in M_1_@M_12_; accordingly, the hollow icosahedral kernel is unlikely to arise in the initial stage of the nanocluster growth. Besides, the hollow M_12_ kernel is also less likely to originate from its non-hollow counterpart because the 12 metal···metal interactions make it difficult to extract the innermost metal atom out. In this context, the origin of such hollow icosahedral kernels remains a mystery.

In this work, based on the Au-alloying-induced nanocluster transformation from M_40_(SR)_24_ to M_44_(SR)_30_ (M = Au/Ag/Cu), a reasonable avenue for the generation of hollow icosahedral M_12_ kernels has been mapped out, i.e., the kernel collapse from several small nano-building blocks to an integrated hollow icosahedron. The proposed avenue might serve as a common approach in constructing metallic kernels of nanoclusters and nanoparticles (not limited to the M_12_ structure).

## Results

### Structural anatomy of M_40_(SR)_24_ and M_44_(SR)_30_ nanoclusters

**Fig. 1:**
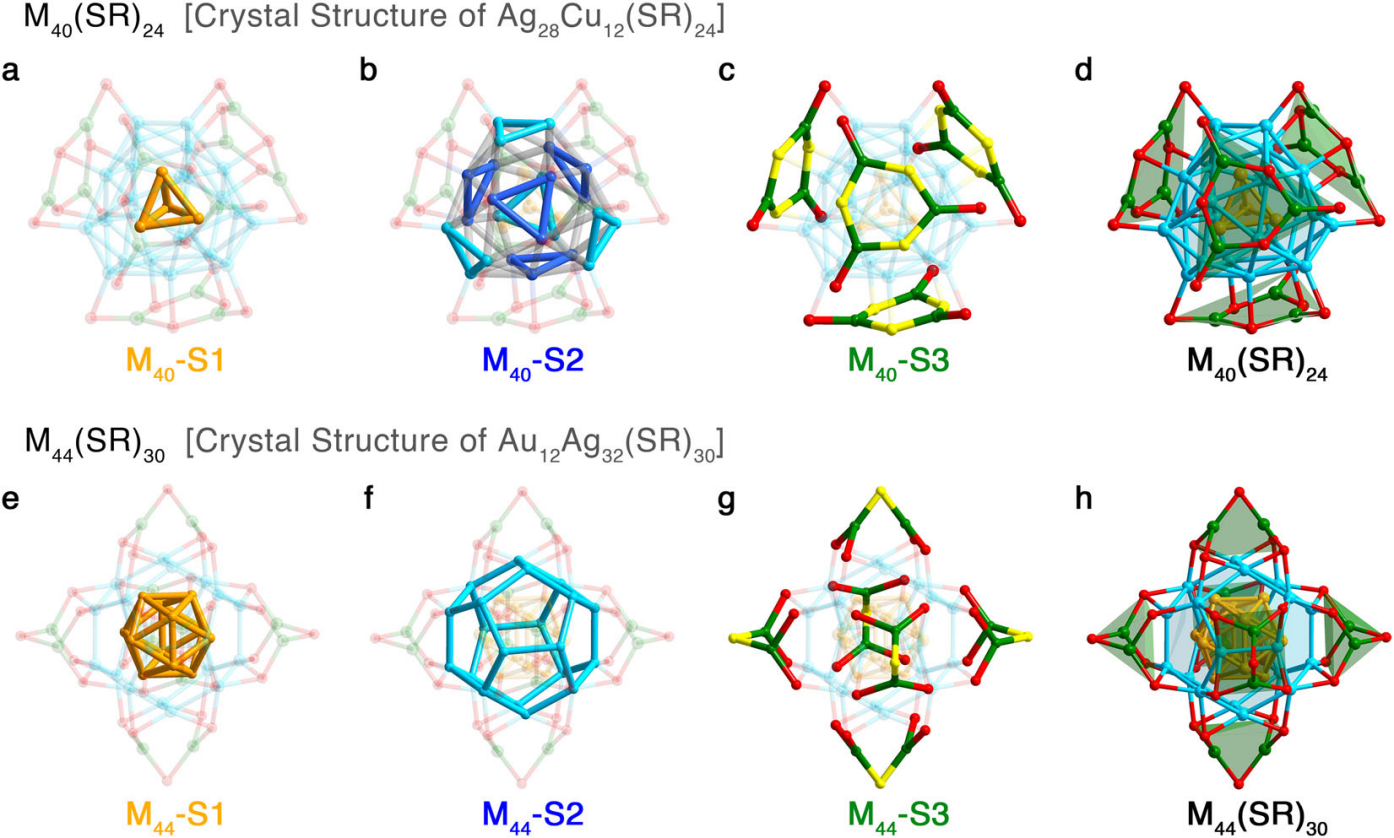
Structure anatomies of M_40_(SPhCl_2_)_24_ and M_44_(SPhCl_2_)_30_ (M = Au/Ag/Cu). **a**–**d** Structure anatomy of M_40_(SPhCl_2_)_24_: **a** M_40_-S1: the tetrahedral M_4_ kernel; **b** M_40_-S2: the M_24_ shell; **c** M_40_-S3: the Cu_12_(SR)_24_ surface; and **d** The overall structure of M_40_(SPhCl_2_)_24_. **e**–**h** Structure anatomy of M_44_(SPhCl_2_)_30_. **e** M_44_-S1: the hollow icosahedral M_12_ kernel; **f** M_44_-S2: the M_20_ shell; **g** M_40_-S3: the M_12_(SR)_30_ surface; and **h** The overall structure of M_44_(SPhCl_2_)_30_. Color labels: orange/light blue/blue/green, Ag or Cu atoms at different positions; red, S. All C and H atoms are omitted for clarity.

For the clarity of the structural transformation and the corresponding kernel collapse, the nanocluster structures involved in this work are first discussed (Fig. 1) —

(i) M_40_(SR)_24_ (M = Au/Ag/Cu; SR = SPhCl_2_): the M_40_(SR)_24_ nanoclusters start from the bi-metallic Ag_28_Cu_12_(SR)_24_^57^. Figure 1a–d depict the structure anatomy of Ag_28_Cu_12_(SR)_24_. Ag_28_Cu_12_(SR)_24_ adopts a three-shell configuration, in a form of Ag_4_(M_40_-S1)@Ag_24_(M_40_-S2)@4*Cu_3_(SR)_6_(M_40_-S3). The innermost Ag_4_ is in tetrahedral (Fig. 1a). The 24 Ag atoms on M_40_-S2 can be divided into two categories, and each of 12 Ag atoms constitute four Ag_3_ triangles (Fig. 1b). The Ag atoms highlighted in dark blue connect with inward SR ligands on M_40_-S3 (Fig. 1c, highlighted in yellow); in contrast, the Ag atoms in light blue links outward SR ligands (highlighted in red) on M_40_-S3.
(ii) M_44_(SR)_30_ (M = Au/Ag/Cu; SR = SPhCl_2_): the Au_12_Ag_32_(SR)_30_ nanocluster is adopted to analyze the structure of M_44_(SR)_30_^58^. Au_12_Ag_32_(SR)_30_ also has a three-shell configuration: Au_12_(M_44_-S1)@Ag_20_(M_44_-S2)@6*Ag_2_(SR)_5_(M_44_-S3). Of note, the Au_12_ kernel is a hollow icosahedron (Fig. 1e).

### Au-alloying-induced transformation from M_40_(SR)_24_ to M_44_(SR)_30_

**Fig. 2:**
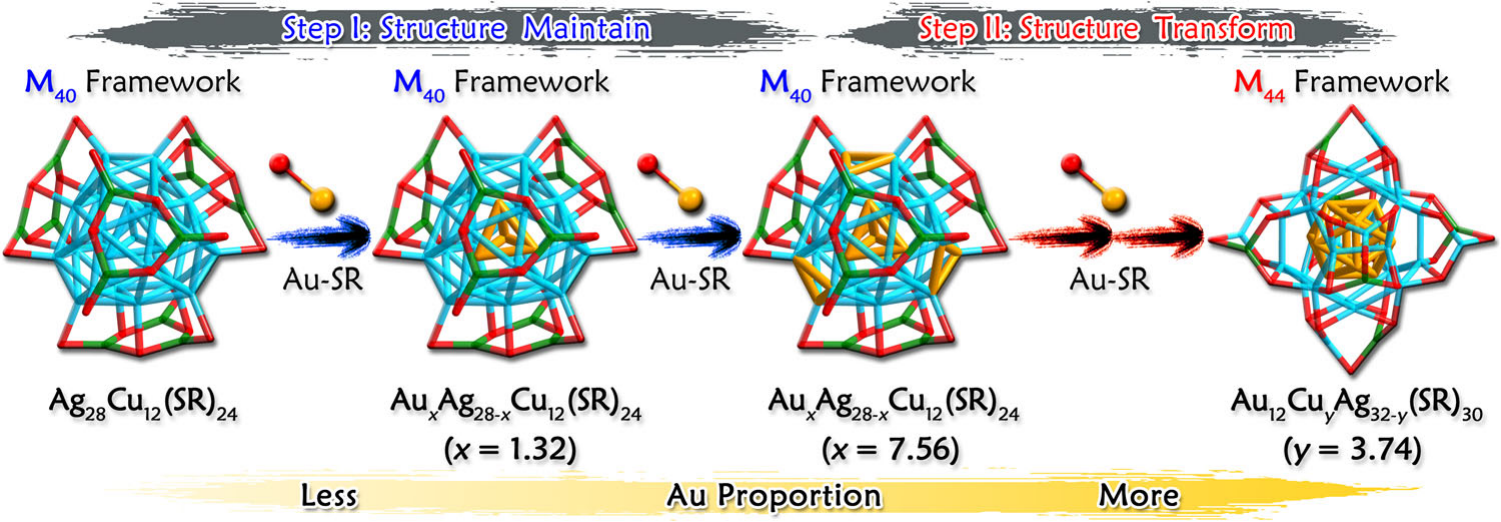
Illustration of Au-alloying-induced structural evolution. Step I contains the Au-doping processes from Ag_28_Cu_12_(SR)_24_ to Au_*x*_Ag_28-*x*_Cu_12_(SR)_24_ (*x* = 1.32) and then to Au_*x*_Ag_28-*x*_Cu_12_(SR)_24_ (*x* = 7.56), in which processes the M_40_(SR)_24_ framework retains. Step II is the Au-doping process from Au_*x*_Ag_28-*x*_Cu_12_(SR)_24_ (*x* = 7.56) to Au_12_Cu_*y*_Ag_32-*y*_(SR)_30_ (*y* = 3.74), in which process the M_40_(SR)_24_ framework transforms into M_44_(SR)_30_ (M = Au/Ag/Cu). The Au proportion gradually increases with the nanocluater evolution from Ag_28_Cu_12_(SR)_24_ to Au_*x*_Ag_28-*x*_Cu_12_(SR)_24_ (*x* = 1.32), Au_*x*_Ag_28-*x*_Cu_12_(SR)_24_ (*x* = 7.56), and Au_12_Cu_*y*_Ag_32-*y*_(SR)_30_ (*y* = 3.74). Color labels: light blue, Ag; orange, Au; green, Cu; red, S. All C and H atoms are omitted for clarity.

The Au-alloying structural transformation started from the bi-metallic Ag_28_Cu_12_(SR)_24_ (Fig. 2). The slight Au alloying on Ag_28_Cu_12_(SR)_24_ resulted in a tri-metallic Au_*x*_Ag_28-*x*_Cu_12_(SR)_24_ (*x* = 1.32) nanocluster, wherein the tetrahedral Ag_4_ kernel of Ag_28_Cu_12_(SR)_24_ was partially alloyed by the incorporated Au (Fig. 2 and Supplementary Fig. 1). When more Au heteroatoms were doped into M_40_(SR)_24_ (Au_*x*_Ag_28-*x*_Cu_12_(SR)_24_, *x* = 7.56; Fig. 2), all sites of the tetrahedron were entirely occupied by Au; besides, the redundant Au heteroatoms were further arranged onto M_40_-S2, invading the Ag sites that related to outward SR thiols (light blue triangles in Fig. 1b and Supplementary Figs. 2–3). Of note, throughout the abovementioned Au-alloying processes the M_40_(SR)_24_ framework retained. Furthermore, the overdose of Au heteroatom induced the structural transformation from M_40_(SR)_24_ to M_44_(SR)_30_. Structurally, from the crystal structure of Au_12_Cu_*y*_Ag_32-*y*_(SR)_30_ (*y* = 0–6; Avg. 3.74; Fig. 2 and Supplementary Fig. 4), the M_44_(SR)_30_ nanocluster reached its stable state when Cu atoms only occupied the M_44_-S3. The corresponding bond lengths in M_40_(SR)_24_ (including Ag_28_Cu_12_(SR)_24_, Au_*x*_Ag_28-*x*_Cu_12_(SR)_24_ (*x* = 1.32), Au_*x*_Ag_28-*x*_Cu_12_(SR)_24_ (*x* = 7.56), and Au_4_Ag_24_Cu_12_(SR)_24_) or M_44_(SR)_30_ (including Au_12_Ag_32_(SR)_30_ and Au_12_Cu_*y*_Ag_32-*y*_(SR)_30_, *y* = 3.74) nanoclusters were compared in detail (Supplementary Figs. 5–6 and Supplementary Tables 1–2).

Despite our repeated best efforts to obtain the crystal structure of Au_12_Cu_*y*_Ag_32-*y*_(SR)_30_, its perfect crystal data remains unavailable. Herein, for acquiring an excellent crystal data of Au_12_Cu_*y*_Ag_32-*y*_(SR)_30_, we promoted the co-crystallization between the M_44_(SR)_30_ and a small-sized M_40_(SR)_24_ nanoclusters (Supplementary Figs. 7–9). Fortunately, the structures of both two nanoclusters were excellently determined (Au_4_Ag_24_Cu_12_(SR)_24_ and Au_12_Cu_*y*_Ag_32-*y*_(SR)_30_ (*y* = 3.74)) although both displayed strongly negative valence state (i.e., “−4”). In this context, the presence of (PPh_4_)^+^ cations neutralized the electrostatic repulsion between these clusters (Supplementary Fig. 8) and thus promoted the nanocluster co-crystallization, which was different from previously reported co-crystallized nanocluster cases with both “0” or opposite valence states^59–62^. In the crystal lattice of the co-crystallized nanoclusters, three types of nanoclusters were observed (Supplementary Fig. 7) — *L*-Au_4_Ag_24_Cu_12_(SR)_24_ (A*α*, A*β*), *R*-Au_4_Ag_24_Cu_12_(SR)_24_ (B*α*, B*β*), and Au_12_Cu_*y*_Ag_32-*y*_(SR)_30_ (*y* = 3.74; C*α*, C*β*), among which the *α*- and *β*-nanoclusters were identical, but arranged in different rotation angles (Supplementary Fig. 7a–c). In contrast to the crystallization of homogeneous nanoclusters, which are typically packed into superlattices with simple translational symmetry, such as ABAB or ABCABC packing pattern^40^, the (AuAgCu)_40_ and (AuAgCu)_44_ nanoclusters were packed with a more complex pattern (Supplementary Fig. 7d–f). From the *x*-axis view, the clusters were packed with an A*α*-B*α*-C*α*#A*β*-B*β*-C*β* pattern. *α*- and *β*-nanoclusters were arranged separately along the *z*-direction, giving rise to *α*- and *β*-cluster lines (Supplementary Fig. 7f). In either cluster line, the adjacent three nanoclusters constituted a repetitive unit, A*α*-B*α*-C*α* or A*β*-B*β*-C*β*, which was labeled by red or black frames, respectively. Such repetitive units were also observed from *y*-axis and *z*-axis views (Supplementary Fig. 7).

The optical absorptions of the obtained nanocluster crystals (dissolved in CH_2_Cl_2_) were compared (Supplementary Fig. 10). Along with the Au-alloying process from Ag_28_Cu_12_(SR)_24_ to Au_*x*_Ag_28-*x*_Cu_12_(SR)_24_ (*x* = 1.32) and Au_*x*_Ag_28-*x*_Cu_12_(SR)_24_ (*x* = 7.56), there was no significant alteration of the optical absorptions (405, 465, and 555 nm). By contrast, when the nanocluster template transformed from M_40_(SR)_24_ to M_44_(SR)_30_, these absorptions shifted to 390, 490, and 595 nm immediately (Supplementary Fig. 10), demonstrating the remarkable change over electronic structures with the template transformation. Time-dependent UV-vis of the transformation from Ag_28_Cu_12_(SR)_24_ to Au_*x*_Ag_28-*x*_Cu_12_(SR)_24_ and then to Au_12_Cu_*y*_Ag_32-*y*_(SR)_30_ were performed to track the cluster transformation (Supplementary Fig. 11a). The changes of UV-vis contained two stages: (stage 1, from Ag_28_Cu_12_(SR)_24_ to Au_*x*_Ag_28-*x*_Cu_12_(SR)_24_) eight isoabsorption points at 360, 385, 420, 445, 485, 550, 570, and 640 nm were observed (Supplementary Fig. 11b); (stage 2, from Au_*x*_Ag_28-*x*_Cu_12_(SR)_24_ to Au_12_Cu_*y*_Ag_32-*y*_(SR)_30_) three isoabsorption points at 400, 485, and 510 nm were detected (Supplementary Fig. 11c). The observation of these isoabsorption points suggested that the overall cluster transformation was a proportional conversion. Accordingly, both transformations from Ag_28_Cu_12_(SR)_24_ to Au_*x*_Ag_28-*x*_Cu_12_(SR)_24_ and from Au_*x*_Ag_28-*x*_Cu_12_(SR)_24_ to Au_12_Cu_*y*_Ag_32-*y*_(SR)_30_ followed an “intramolecular rearrangement” approach, but not an “intermolecular decomposition-recombination” approach.

Besides, along with the Au-alloying process, the thermal stability of nanoclusters was enhanced. As shown in Supplementary Fig. 12, UV-vis characteristic absorptions of the Ag_28_Cu_12_(SR)_24_ nanocluster (dissolved in CH_2_Cl_2_) gradually decreased in intensity after 1 h and completely disappeared in ~4 h, indicating degradation. In contrast, the UV-vis absorptions of Au_*x*_Ag_28-*x*_Cu_12_(SR)_24_ (*x* = 1.32) were essentially identical in the first 2 h, and gradually decreased as time went on. Of note, the optical absorptions of Au_*x*_Ag_28-*x*_Cu_12_(SR)_24_ (*x* = 7.56) was almost retained within 24 h, suggesting the enhanced thermal stability of Au_*x*_Ag_28-*x*_Cu_12_(SR)_24_ (*x* = 7.56) over other two M_40_(SR)_24_ nanoclusters. In this context, the sequence of the thermal stability of these three M_40_(SR)_24_ nanoclusters was determined as Au_*x*_Ag_28-*x*_Cu_12_(SR)_24_ (*x* = 7.56) > Au_*x*_Ag_28-*x*_Cu_12_(SR)_24_ (*x* = 1.32) > Ag_28_Cu_12_(SR)_24_; that is, increasing the Au-doping amount in nanoclusters was in favor of preparing M_40_(SR)_24_ with higher thermal stability.

Electrospray ionization mass spectrometry (ESI-MS) was then performed on nanocluster crystals (dissolved in CH_2_Cl_2_), and the mass results confirmed the compositions of these “−4”-charged M_40_(SR)_24_ and M_44_(SR)_30_ nanoclusters (Supplementary Fig. 13). Besides, the in-situ Au-alloying process and the nanocluster template transformation were tracked by exploiting the ESI-MS (Supplementary Figs. 14–18). At the very beginning process (1–2 min in Supplementary Fig. 14), only Au_*x*_Ag_28-*x*_Cu_12_(SR)_24_ nanoclusters were detected (Fig. 2), corresponding to the Au-doping process from Ag_28_Cu_12_(SR)_24_ to Au_*x*_Ag_28-*x*_Cu_12_(SR)_24_ (*x* = 1.32) and Au_*x*_Ag_28-*x*_Cu_12_(SR)_24_ (*x* = 7.56). The further Au-alloying induced both the Au component growth in M_40_(SR)_24_ and the template transformation from M_40_(SR)_24_ to M_44_(SR)_30_ (3–6 min in Supplementary Fig. 14). Finally, only Au_12_Cu_*y*_Ag_32-*y*_(SR)_30_ nanoclusters could be observed (7–8 min in Supplementary Fig. 14), which suggested the complete transformation of nanoclusters. Of note, the Au_12_Ag_32-*y*_Cu_*y*_(SR)_30_ would stable at *y* = 3 or 4 once generated, matching with the crystal structure of Au_12_Cu_*y*_Ag_32-*y*_(SR)_30_ (*y* = 3.74). Energy-dispersive X-ray spectroscopy (EDS) mapping and X-ray photoelectron spectroscopy (XPS) were conducted to confirm the Au-alloying process (Supplementary Figs. 19–28).

### Kernel transformation from tetrahedron to hollow icosahedron

**Fig. 3:**
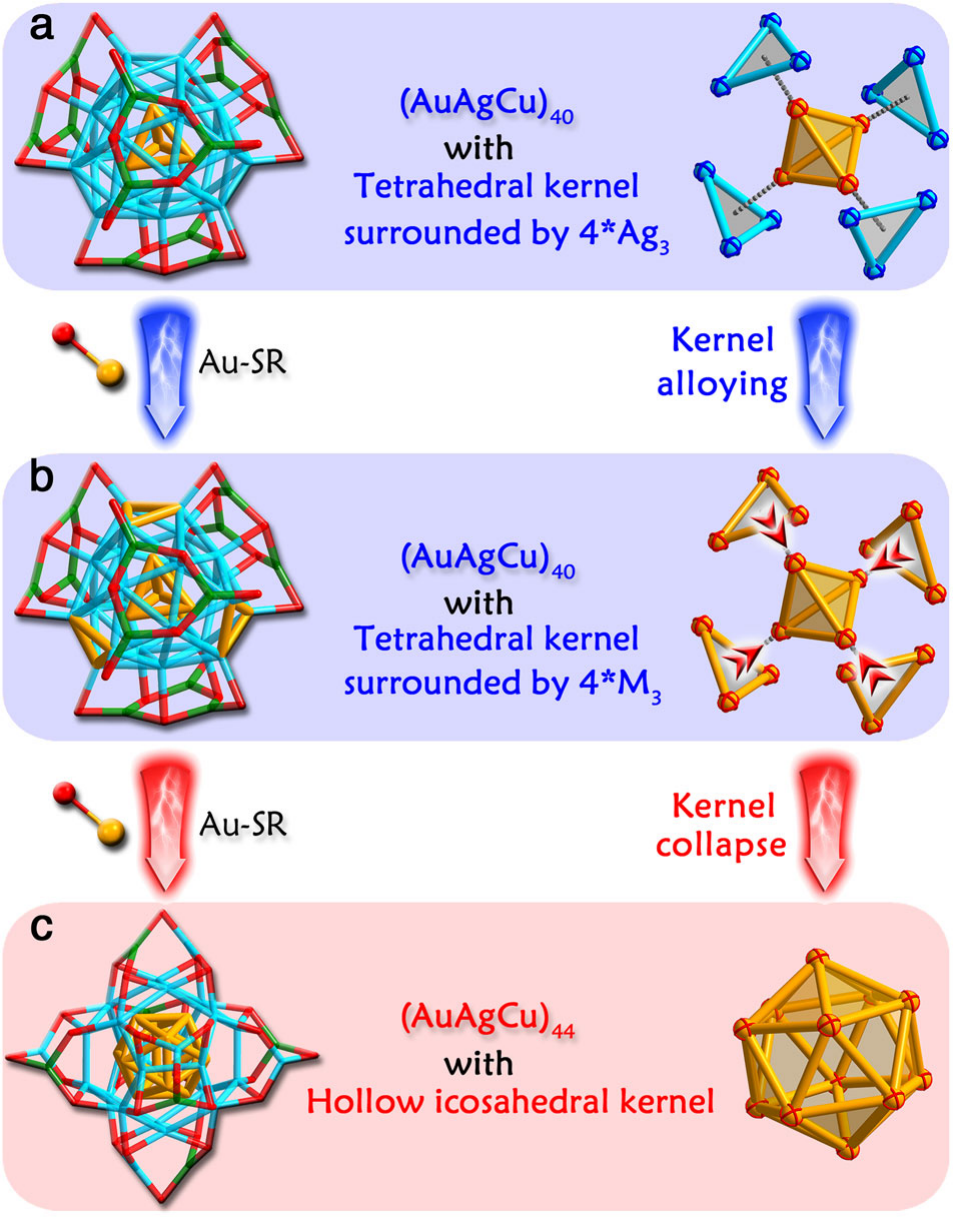
Kernel transformation from tetrahedron to hollow icosahedron. **a** Crystal structure of Au_*x*_Ag_28-*x*_Cu_12_(SR)_24_ (*x* = 1.32) and its tetrahedral M_4_ (M = Au/Ag) kernel surrounded by four Ag_3_ triangles. **b** Crystal structure of Au_*x*_Ag_28-*x*_Cu_12_(SR)_24_ (*x* = 7.56) and its tetrahedral Au_4_ kernel surrounded by four M_3_ (M = Au/Ag) triangles. The red arrows represent the trend of kernel collapse from “tetrahedral Au_4_ + 4^∗^M_3_” to “hollow icosahedral Au_12_”. **c** Crystal structure of Au_12_Cu_*y*_Ag_32-*y*_(SR)_30_ (*y* = 3.74) and its hollow icosahedral Au_12_ kernel. Color labels: light blue, Ag; orange, Au; green, Cu; red, S. All C and H atoms are omitted for clarity.

Figure 3 depicts the kernel collapse from the “tetrahedral Au_4_ + 4*M_3_” to “hollow icosahedral Au_12_” induced by the Au alloying. Specifically, the initial Au-doping process transported the Au heteroatoms to the tetrahedral kernel, converting the Ag_4_ kernel to the alloyed Au_*x*_Ag_4-*x*_ and the final Au_4_ (Fig. 3a). The further Au-alloying sites on M_40_-S2 predominantly located at the four M_3_ triangles that adhered to a vertex-to-face relationship to the tetrahedral Au_4_ kernel (Fig. 3b and Supplementary Fig. 3c); in contrast, the other four triangles on M_40_-S2, following a face-to-face relationship to the Au_4_ tetrahedral kernel, maintained unalloyed as Ag_3_ (Supplementary Fig. 3d). For easily distinguishing these M_3_ positions, we define these Au_3_ positions as “stable location” (Supplementary Fig. 3c) and “unstable location” (Supplementary Fig. 3d). However, the Au doping on stable locations is simply concluded from the crystallography, and the Au positions may change throughout the crystallization process. From ESI-MS results (Supplementary Fig. 14), a maximum of 18–19 Au heteroatoms could be doped into the M_40_ cluster framework, >16 positions from the M_4_ kernel and 4*M_3_ stable locations; accordingly, there are other Ag positions in M_40_ that could be occupied by the introduced Au. X-ray absorption fine structure spectroscopy (XAFS) measurements were then performed for grasping the in-situ Au-doping process (Supplementary Figs. 29 and 30 and Supplementary Tables 3–4). The XAFS results demonstrated that the introduced Au occupied the innermost M_4_ tetrahedron first, and then substituted the Ag atoms in unstable locations, different from the crystal results wherein the unstable locations were maintained as undoped Ag throughout. We further crystallized this cluster sample and the crystal data suggested the Au heteroatoms on stable locations (i.e., Au_*x*_Ag_28-*x*_Cu_12_(SR)_24_, *x* = 7.76), demonstrating the intracluster Au–Ag metal exchange throughout the crystallization. In this context, we made some speculations on mass signals (Supplementary Fig. 14): the introduced Au heteroatoms occupied the innermost tetrahedron first, and then substituted Ag atoms on M_40_-S2 randomly; the mass signals i represented the dominant Au-occupation in stable locations, whereas the signals ii represented the unstable locations, resulting in two groups of signals in the 3-min mass spectrum (Supplementary Fig. 14). In the 3-min sample, the M_40_ with Au-occupation in unstable locations might be the main product by referring XAFS results. Then, the M_40_ clusters of signals ii would transform to M_44_ clusters of signals iii, and then decomposed due to their instability. By comparison, the M_40_ clusters of signals i were continually doped by Au and transformed to M_44_ clusters of signals iv finally. In this context, the driving force for the transformation from M_40_ to M_44_ was determined as the Au-alloying at unstable locations, which rendered the M_40_ nanoclusters unstable molecules and triggered the kernel collapse from several small nano-building blocks to an integrated hollow icosahedron.

Significantly, the further Au-alloying induced the transformation from M_40_(SR)_24_ to M_44_(SR)_30_, among which process the hollow icosahedral Au_12_ was generated (Fig. 3b, c). Structurally, the pre-transformed Au_*x*_Ag_28-*x*_Cu_12_(SR)_24_ possesses a “tetrahedral Au_4_ + 4*M_3_” kernel (M = Au/Ag with a high Au proportion). Upon the nanocluster conversion, the M_3_ triangles collapsed inward to the Au_4_ tetrahedron, and finally rearranged into the hollow icosahedral Au_12_ kernel in Au_12_Cu_*y*_Ag_32-*y*_(SR)_30_ (Fig. 3c). Of note, there are 16 metal atoms in the “tetrahedral M_4_ + 4*M_3_” kernel while the icosahedral kernel only contains 12 metal atoms; in this context, a structural rearrangement occurred in this structural and kernel transformation (indeed, the “kernel+surface” configurations between M_40_(SR)_24_ and M_44_(SR)_30_ nanoclusters are different). However, due to the existence of several isoabsorption points in the UV-vis spectra, the structure transformation from M_40_(SR)_24_ to M_44_(SR)_30_ should follow an “intramolecular rearrangement” approach, but not an “intermolecular decomposition-recombination” approach. Accordingly, it is reasonable to conjecture the formation of icosahedral M_12_ in M_44_(SR)_30_ as the kernel collapse from “tetrahedral Au_4_ + 4*M_3_”. Besides, all sites in the hollow icosahedron are fully occupied by Au (i.e., Au_12_); in vivid contrast, the non-hollow M_1_@M_12_ kernels of previously alloy clusters are always partially occupied by two or more types of metals. We proposed that the complete Au occupation of the hollow icosahedron resulted from the kernel collapse in which process only the collapse of Au atom to Au_4_ was the most energetically favorable.

This avenue (i.e., kernel collapse) is of great importance since it maps out a reasonable avenue for the generation of the hollow icosahedral M_12_ kernel in metal nanoclusters. Besides, the kernel collapse might be a very common approach in constructing metallic kernels of nanoclusters and nanoparticles (not limited to the hollow icosahedron, but also compliant to other configurations such as non-hollow icosahedron, FCC/BCC kernels, etc.), because the routine growth of several large-sized nanoclusters shell-by-shell should be not that energetically favorable. We also note that the kernel collapse should not be the unique approach for the generation of hollow icosahedra (or other structures) in metal nanoclusters and nanoparticles; other approaches may also exist and are still worth mapping out.

## Discussion

In summary, on the basis of the Au-alloying-induced transformation from M_40_(SR)_24_ to M_44_(SR)_30_ (M = Au/Ag/Cu), a reasonable avenue—kernel collapse—for the generation of the hollow icosahedral M_12_ kernel in metal nanoclusters has been mapped out. The Au alloying on Ag_28_Cu_12_(SR)_24_ produced template-maintained Au_*x*_Ag_28-*x*_Cu_12_(SR)_24_ (*x* = 1.32), Au_*x*_Ag_28-*x*_Cu_12_(SR)_24_ (*x* = 7.56), and template-transformed Au_12_Cu_*y*_Ag_32-*y*_(SR)_30_ (*y* = 3.74) step by step, accompanying with which processes the cluster kernel stepwisely evolved from “tetrahedral Ag_4_ + 4*Ag_3_” to “tetrahedral Au_4_ + 4*Ag_3_”, then to “tetrahedral Au_4_ + 4*Au_3_”, and finally to “hollow icosahedral Au_12_”. The entire process was tracked by ESI-MS, and the crystal structures of the key nodes (altogether five crystal structures) have been determined. Overall, this work presents a reasonable avenue for comprehending the generation of hollow icosahedra in metal nanoclusters, and the “structure collapse” might be a very common approach for constructing kernel structures (not limited to the hollow icosahedron) in the size growth of nanoclusters and nanoparticles.

## Methods

### Materials

All reagents were purchased from Sigma-Aldrich and used without further purification: silver nitrate (AgNO_3_, 99%, metal basis), tetrachloroauric (III) acid (HAuCl_4_·3H_2_O, 99.99% metal basis), copper(II) acetylacetonate (Cu(O_2_C_5_H_7_)_2_, 99%, metal basis), 2,4-dichlorobenzenethiol (HSPhCl_2_, 99%), sodium borohydride (NaBH_4_, 97%), tetraphenylphosphonium bromide ((PPh_4_)Br, 95%), dichloromethane (CH_2_Cl_2_, HPLC, Sigma-Aldrich), methanol (CH_3_OH, HPLC, Sigma-Aldrich), N,N-dimethylformamide (DMF, HPLC, Sigma-Aldrich), hexane (C_6_H_6_, HPLC, Sigma-Aldrich), and ethyl ether ((CH_3_CH_2_)O, HPLC, Sigma-Aldrich).

### Synthesis of Au(I)-SPhCl_2_

For the Au(I)-SPhCl_2_ complexes synthesis, HAuCl_4_·3H_2_O (1 mmol) was dissolved in 5 mL CH_3_OH, and 2,4-dichlorobenzenethiol (500 μL, 4 mmol) was dissolved in 5 mL CH_3_OH and added drop-wise to the solution under vigorously stirring (~1200 rpm). After reacted for 15 min, the resulting precipitate was washed several times with hexane. Then the final product was used directly.

### Synthesis of [Ag_28_Cu_12_(SPhCl_2_)_24_]^4−^

The Ag_28_Cu_12_(SPhCl_2_)_24_ was prepared by a literature method reported by the Zheng group with some modification^57^. Specifically, 60 mg of Cu(O_2_C_5_H_7_)_2_ was dissolved in 5 mL of CH_3_OH and 15 mL of CH_2_Cl_2_, to which 60 mg AgNO_3_ (dissolved in 2 mL of H_2_O) was added. After stirring for 20 min, 100 μL of HSPhCl_2_ was added in, and the reaction further processed for 30 min. Then, 30 mg NaBH_4_ (dissolved in 2 mL of H_2_O) was added in. The reaction was allowed to proceed for 5 h. After that, the aqueous layer was removed, and the mixture in the organic phase was rotavaporated under vacuum. Then 50 mL of CH_3_OH was used to extract the Ag_28_Cu_12_(SPhCl_2_)_24_ nanocluster, to which supernatant 20 mg of (PPh_4_)Br was added in. The precipitate was then washed three times by CH_3_OH. Then the final product, i.e., [Ag_28_Cu_12_(SPhCl_2_)_24_]^4−^(PPh_4_)_4_, was used directly. The yield is 35% based on the Ag element (calculated from the AgNO_3_).

### Syntheses of [Au_*x*_Ag_28-*x*_Cu_12_(SPhCl_2_)_24_]^4−^ (x = 1.32), [Au_*x*_Ag_28-*x*_Cu_12_(SPhCl_2_)_24_]^4−^ (*x* = 7.56), and [Au_12_Cu_*y*_Ag_32-*y*_(SPhCl_2_)_30_]^4−^ nanoclusters

These three nanoclusters were prepared from parallel Au-alloying reactions (in the same condition but were stopped at different times). Specifically, 20 mg of Ag_28_Cu_12_(SPhCl_2_)_24_ was first dissolved in 20 mL of CH_2_Cl_2_ and then 5 mg of Au(I)-SPhCl_2_ complexes was added in. After 2 min, 100 mL of hexane was poured in to pause the reaction; the precipitate was then dissolved in 20 mL of CH_2_Cl_2_ to yield the Au_*x*_Ag_28-*x*_Cu_12_(SPhCl_2_)_24_ (*x* = 1.32). Expanding the reaction time from 2 min to 3 min would produce the Au_*x*_Ag_28-*x*_Cu_12_(SPhCl_2_)_24_ (*x* = 7.56). Expanding the reaction time from 2 min to 8 min would produce the Au_12_Cu_*y*_Ag_32-*y*_(SPhCl_2_)_30_.

### Synthesis of [Au_12_Ag_32_(SPhCl_2_)_30_]^4−^

The Au_12_Ag_32_(SPhCl_2_)_30_ nanocluster was prepared by a literature method reported by the Zheng group^58^.

### Preparation of XAFS samples

In all, 10 mg of Ag_28_Cu_12_(SPhCl_2_)_24_ was dissolved in 10 mL of CH_2_Cl_2_ and then 3 mg of Au(I)-SPhCl_2_ complexes was added in. After 1 min, 200 mL of hexane was poured in to pause the reaction; the precipitate was then dissolved in 5 mL of CH_2_Cl_2_ to yield the Au_*x*_Ag_28-*x*_Cu_12_(SPhCl_2_)_24_ (Sample 1). In total, 10 mg of Ag_28_Cu_12_(SPhCl_2_)_24_ was first dissolved in 10 mL of CH_2_Cl_2_ and then 3 mg of Au(I)-SPhCl_2_ complexes was added in. After 2 min, 200 mL of hexane was poured in to pause the reaction; the precipitate was then dissolved in 5 mL of CH_2_Cl_2_ to yield the Au_*x*_Ag_28-*x*_Cu_12_(SPhCl_2_)_24_ (Sample 2). Single crystals of XAFS Sample 2 (i.e., Au_*x*_Ag_28-*x*_Cu_12_(SPhCl_2_)_24_, *x* = 7.76) were cultivated at room temperature by vapor diffusing the ethyl ether into the DMF solution of nanoclusters.

### Crystallization of [Ag_28_Cu_12_(SPhCl_2_)_24_]_1_(PPh_4_)_4_, [Au_*x*_Ag_28-*x*_Cu_12_(SPhCl_2_)_24_]_1_(PPh_4_)_4_ (*x* = 1.32), [Au_*x*_Ag_28-*x*_Cu_12_(SPhCl_2_)_24_]_1_(PPh_4_)_3_ (*x* = 7.56), [Au_*x*_Ag_28-*x*_Cu_12_(SPhCl_2_)_24_]_1_(PPh_4_)_4_ (*x* = 7.76), [Au_12_Cu_*y*_Ag_32-*y*_(SPhCl_2_)_30_]^4−^ and [Au_12_Ag_32_(SPhCl_2_)_30_]_1_[N(C_4_H_9_)_4_]_4_ nanoclusters

Single crystals of these nanoclusters were cultivated at room temperature by vapor diffusing the ethyl ether into the DMF solution of them. After 21 days, black crystals were collected, and the structures of these nanoclusters were determined. The CCDC number of [Ag_28_Cu_12_(SPhCl_2_)_24_]_1_(PPh_4_)_4_ is 2009375; the CCDC number of [Au_*x*_Ag_28-*x*_Cu_12_(SPhCl_2_)_24_]_1_(PPh_4_)_4_ (*x* = 1.32) is 2009456; the CCDC number of [Au_*x*_Ag_28-*x*_Cu_12_(SPhCl_2_)_24_]_1_(PPh_4_)_3_ (*x* = 7.56) is 2009457; the CCDC number of [Au_*x*_Ag_28-*x*_Cu_12_(SPhCl_2_)_24_]_1_(PPh_4_)_4_ (*x* = 7.76) is 2083130; the CCDC number of [Au_12_Cu_*y*_Ag_32-*y*_(SPhCl_2_)_30_]^4−^ is 2009378; and the CCDC number of [Au_12_Ag_32_(SPhCl_2_)_30_]_1_[N(C_4_H_9_)_4_]_4_ is 1936551. Of note, the perfect crystal data of [Au_12_Cu_*y*_Ag_32-*y*_(SPhCl_2_)_30_]^4−^ remained unavailable despite our repeated efforts, and we only got its kernel structure (i.e., Au_12_Cu_*y*_Ag_32-*y*_S_30_) while the peripheral C, H, and Cl atoms were hard to determine.

### Co-crystallization between M_40_(SPhCl_2_)_24_ and M_44_(SPhCl_2_)_30_ nanoclusters ([Au_4_Ag_24_Cu_12_(SR)_24_]_2_[Au_12_Cu_*y*_Ag_32-*y*_(SR)_30_]_1_, *y* = 3.74)

In all, 20 mg of Au_12_Cu_*y*_Ag_32-*y*_(SPhCl_2_)_30_ (8-min sample in Supplementary Fig. 8) and 20 mg of Au_*x*_Ag_28-*x*_Cu_12_(SPhCl_2_)_30_ (2-min sample in Supplementary Fig. 14) were dissolved in 5 mL of DMF. Single crystals of the co-crystallized nanoclusters were cultivated at room temperature by vapor diffusing the ethyl ether into the DMF solution. After 21 days, black crystals were collected, and the structure of the co-crystallized nanoclusters was determined. The CCDC number of the co-crystallized [Au_4_Ag_24_Cu_12_(SR)_24_]_2_[Au_12_Cu_*y*_Ag_32-*y*_(SR)_30_]_1_ (*y* = 3.74) is 2009377.

### Time-dependent ESI-MS of the Au alloying process on Ag_28_Cu_12_(SPhCl_2_)_24_

In total, 20 mg of Ag_28_Cu_12_(SPhCl_2_)_24_ was firstly dissolved in 20 mL of CH_2_Cl_2_ and then 5 mg of Au(I)-SPhCl_2_ complexes (powder) was added in. The ESI-MS measurement of the reaction was performed every minute.

### X-ray absorption fine structure spectroscopy measurements

XAFS measurements at the Au L3-edge (11919 eV) were performed at the beamline BL14W1 station of the Shanghai Synchrotron Radiation Facility (SSRF), China. The storage ring of the SSRF was working at an energy of 3.5 GeV with an average electron current of 300 mA. The hard X-ray was monochromatized with a Si (311) monochromator. XAFS data were collected in the transmission mode in the energy range from 200 below to 1000 eV above the Au L3-edge. The acquired XAFS data were processed according to the standard procedures using the ARTEMIS module implemented in the IFEFFIT software packages.

### X-ray crystallography

For the crystal date of Ag_28_Cu_12_(SPhCl_2_)_24_, Au_*x*_Ag_28-*x*_Cu_12_(SPhCl_2_)_24_ (*x* = 1.32), Au_12_Cu_*y*_Ag_32-*y*_(SPhCl_2_)_30_, Au_*x*_Ag_28-*x*_Cu_12_(SPhCl_2_)_24_ (*x* = 7.76), and the co-crystallized [Au_4_Ag_24_Cu_12_(SR)_24_]_2_[Au_12_Cu_*y*_Ag_32-*y*_(SR)_30_]_1_ (*y* = 3.74): the data collection for single-crystal X-ray diffraction was carried out on Stoe Stadivari diffractometer under nitrogen flow, using graphite-monochromatized Cu Kα radiation (λ = 1.54186 Å). For the crystal date of Au_*x*_Ag_28-*x*_Cu_12_(SPhCl_2_)_24_ (*x* = 7.56), Au_12_Ag_32_(SPhCl_2_)_30_: the data collection for single crystal X-ray diffraction was carried out on a Bruker Smart APEX II CCD diffractometer under liquid nitrogen flow, using graphite-monochromatized Mo Kα radiation (λ = 0.71069 Å). Data reductions and absorption corrections were performed using the SAINT and SADABS programs, respectively^63^. The structure was solved by direct methods and refined with full-matrix least squares on F^2^ using the SHELXTL software package^64^. All non-hydrogen atoms were refined anisotropically, and all the hydrogen atoms were set in geometrically calculated positions and refined isotropically using a riding model. All crystal structures were treated with PLATON SQUEEZE, and the diffuse electron densities from these residual solvent molecules were removed^65^.

### Characterization

The UV-vis absorption spectra of nanoclusters were recorded using an Agilent 8453 diode array spectrometer. Electrospray ionization mass spectrometry (ESI-MS) measurements were performed by MicrOTOF-QIII high-resolution mass spectrometer. The sample was directly infused into the chamber at 5 μL/min. For preparing the ESI samples, nanoclusters were dissolved in CH_2_Cl_2_ (1 mg/mL) and diluted (*v*/*v* = 1:2) by CH_3_OH. Energy-dispersive X-ray spectroscopy (EDS) mapping of nanoclusters were characterized by SEM (Quanta 400 F). X-ray photoelectron spectroscopy (XPS) measurements were performed on a Thermo ESCALAB 250 configured with a monochromatized Al K*α* (1486.8 eV) 150 W X-ray source, 0.5 mm circular spot size, flood gun to counter charging effects, and analysis chamber base pressure lower than 1 × 10^−9^ mbar.

## Supplementary information

Supplementary Information

## Data Availability

The X-ray crystallographic coordinates for structures reported in this work have been deposited at the Cambridge Crystallographic Data Center (CCDC), under deposition numbers CCDC-2009375, 2009456, 2009457, 2009377, 2009378, and 2083130. These data can be obtained free of charge from the Cambridge Crystallographic Data Centre via www.ccdc.cam.ac.uk/data_request/cif, which has been mentioned in the article.
