# Supplementary material for: A reasonable approach for the generation of hollow icosahedral kernels in metal nanoclusters
Source: Nat Commun. 2021 Oct 26;12:6186. doi: 10.1038/s41467-021-26528-w (PMC8548331; doi:10.1038/s41467-021-26528-w)
Supplement: Supplementary file 1 — Supplementary Information [file 41467_2021_26528_MOESM1_ESM.pdf]

Supplementary Information

A Reasonable Approach for the Generation of Hollow Icosahedral Kernels in Metal Nanoclusters

X. Kang et al.

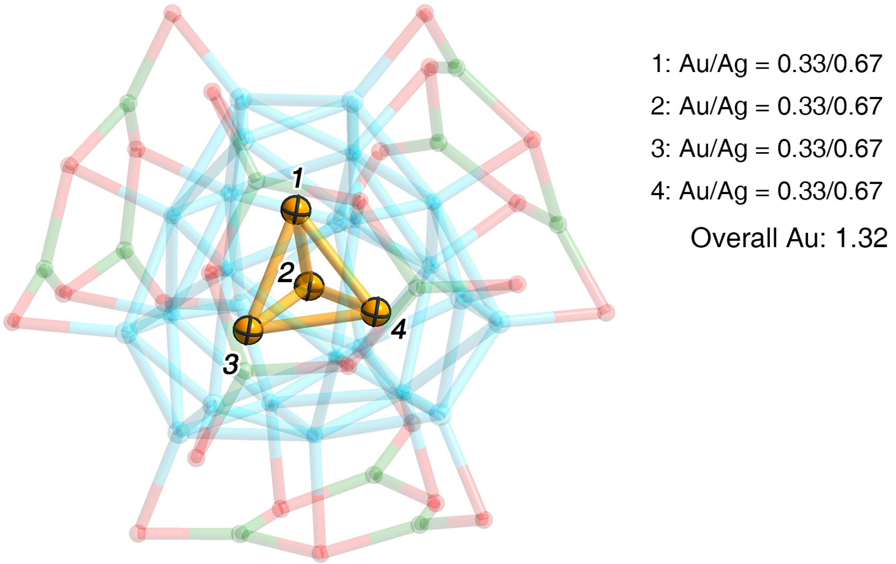

Supplementary Fig. 1 Partial occupation analysis of the $Au_xAg_{28-x}Cu_{12}(SPhCl_2)_{24}$ (x = 1.32) nanocluster. Only the innermost tetrahedral M_4 kernel are co-occupied by Au/Ag. The overall Au occupation number is 1.32. In this context, the molecular formula of this nanocluster is $Au_xAg_{28-x}Cu_{12}(SPhCl_2)_{24}$ (x = 1.32).

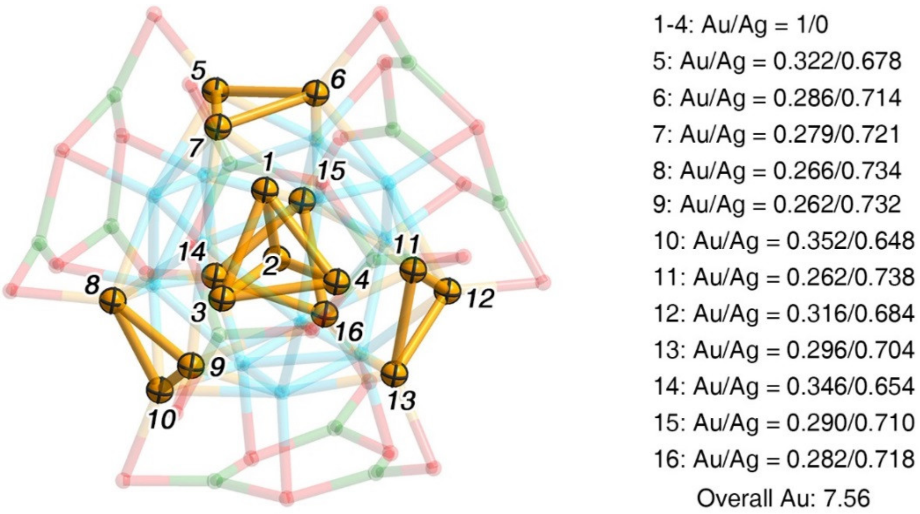

Supplementary Fig. 2 Partial occupation analysis of the $Au_xAg_{28-x}Cu_{12}(SPhCl_2)_{24}$ (x = 7.56) nanocluster. The innermost tetrahedral M_4 kernel is fully occupied by Au. Half of the Ag atoms on the Ag_{24} shell can be substituted by the incorporated Au, resulting in 4 $(Au/Ag)_3$ triangles in this shell. The overall Au occupation number is 7.56. In this context, the molecular formula of this nanocluster is $Au_xAg_{28-x}Cu_{12}(SPhCl_2)_{24}$ (x = 7.56).

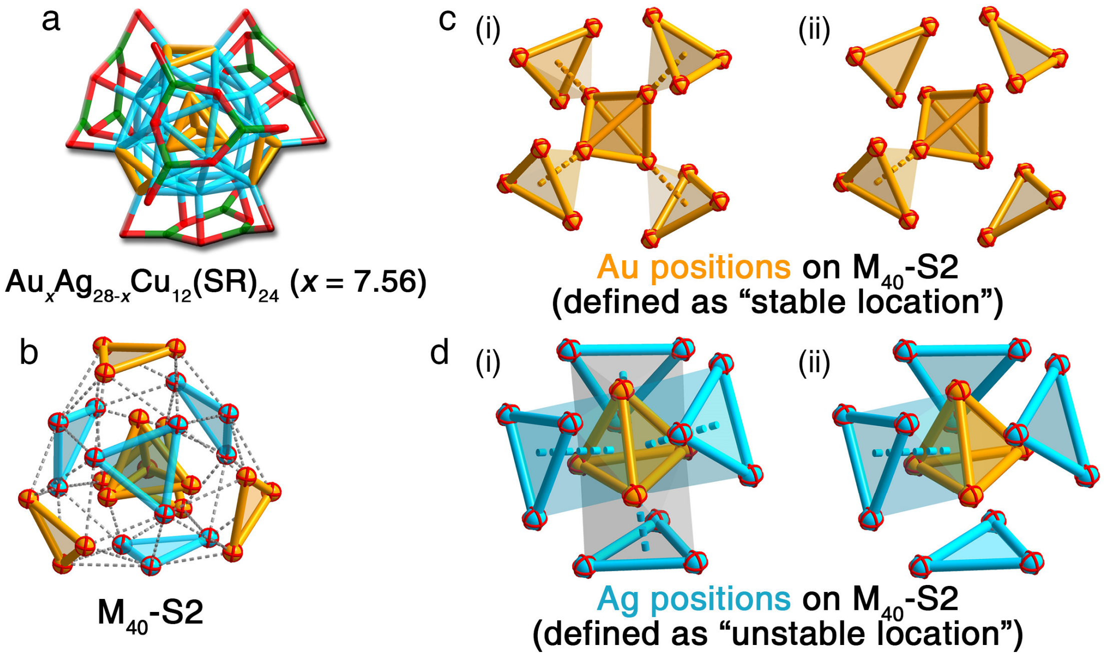

Supplementary Fig. 3 a Crystal structure of $Au_xAg_{28\text{-}x}Cu_{12}(SPhCl_2)_{24}$ (x = 7.56). **b** The shell 2 of $Au_xAg_{28\text{-}x}Cu_{12}(SPhCl_2)_{24}$ (x = 7.56) contains 8 M_3 (M = Au/Ag) triangles including 4 Ag_3 triangles (highlighted in blue) and 4 M_3 triangles (highlighted in orange). **c** The vertex-to-face relationship between the tetrahedral Au_4 kernel and M_3 triangles on shell 2. **d** The face-to-face relationship between the tetrahedral Au_4 kernel and Ag_3 triangles on shell 2. For easily distinguishing these M_3 positions, we define these Au_3 positions as **c** "*stable location*" and **d** "*unstable location*".

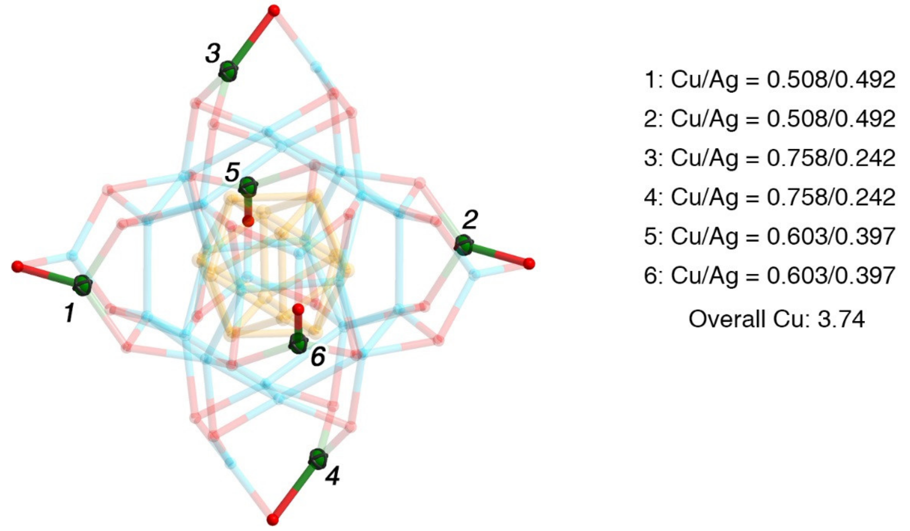

Supplementary Fig. 4 Partial occupation analysis of the $Au_{12}Cu_yAg_{32\text{-}y}(SPhCl_2)_{30}$ (y = 3.74) nanocluster. Half of Ag atoms at the outermost 6*[$Ag_2(SR)_6$] shells can be substituted by Cu, which are labelled with 1-6 in the figure. The overall Au occupation number is 3.74. In this context, the molecular formula of this nanocluster is $Au_{12}Cu_yAg_{32\text{-}y}(SPhCl_2)_{30}$ (y = 3.74).

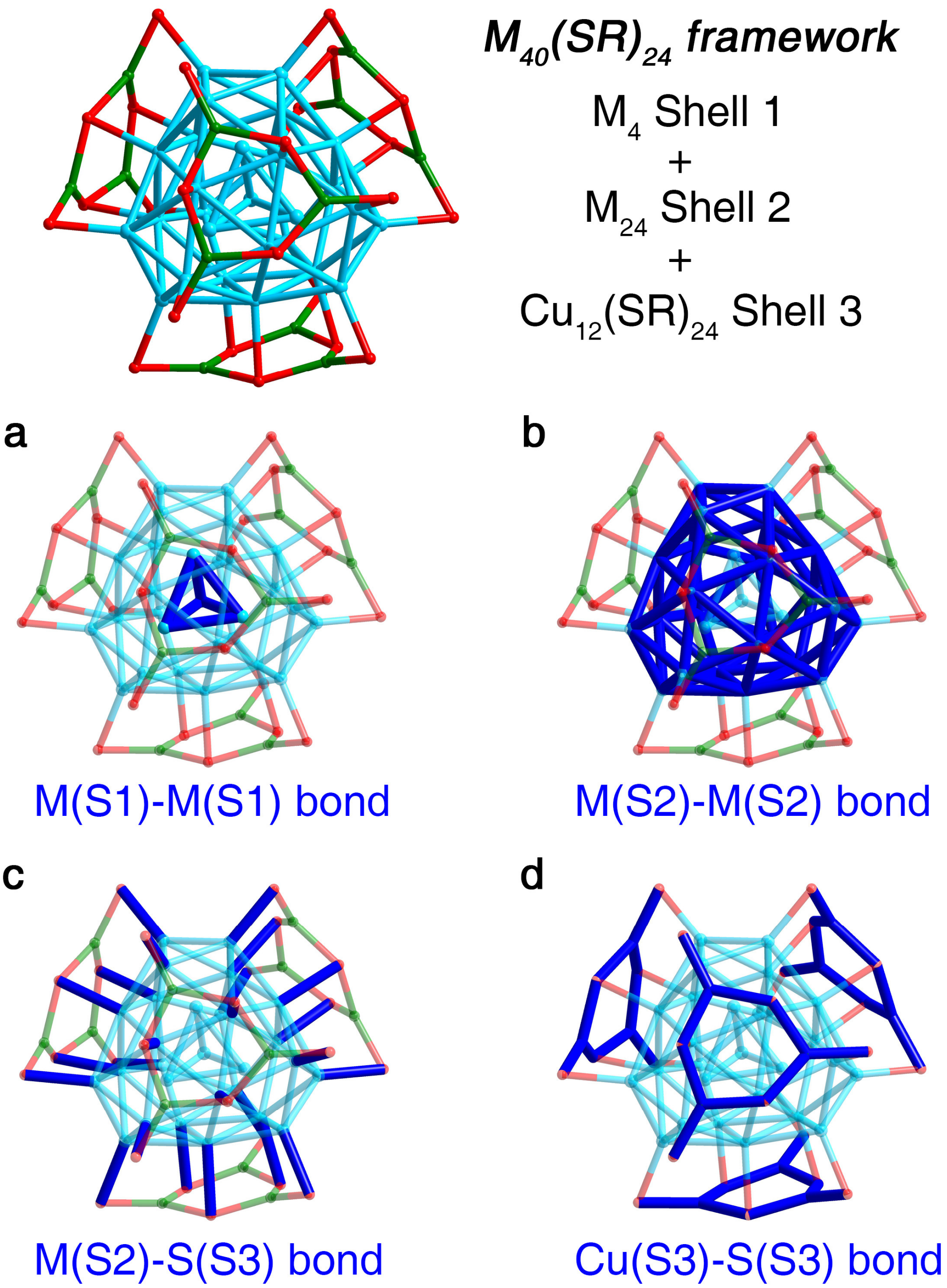

Supplementary Fig. 5 Comparison of the bond lengths (corresponding to Supplementary Table 1) among different $M_{40}(SR)_{24}$ (M = Au/Ag/Cu; SR = $SPhCl_2$) nanoclusters. **a** M(S1)-M(S1) bonds (M = Au/Ag). **b** M(S2)-M(S2) bonds (M = Au/Ag). **c** M(L2)-S(L3) bonds (M = Au/Ag). **d** Cu(S3)-S(S3) bonds. The compared bonds are highlighted in blue.

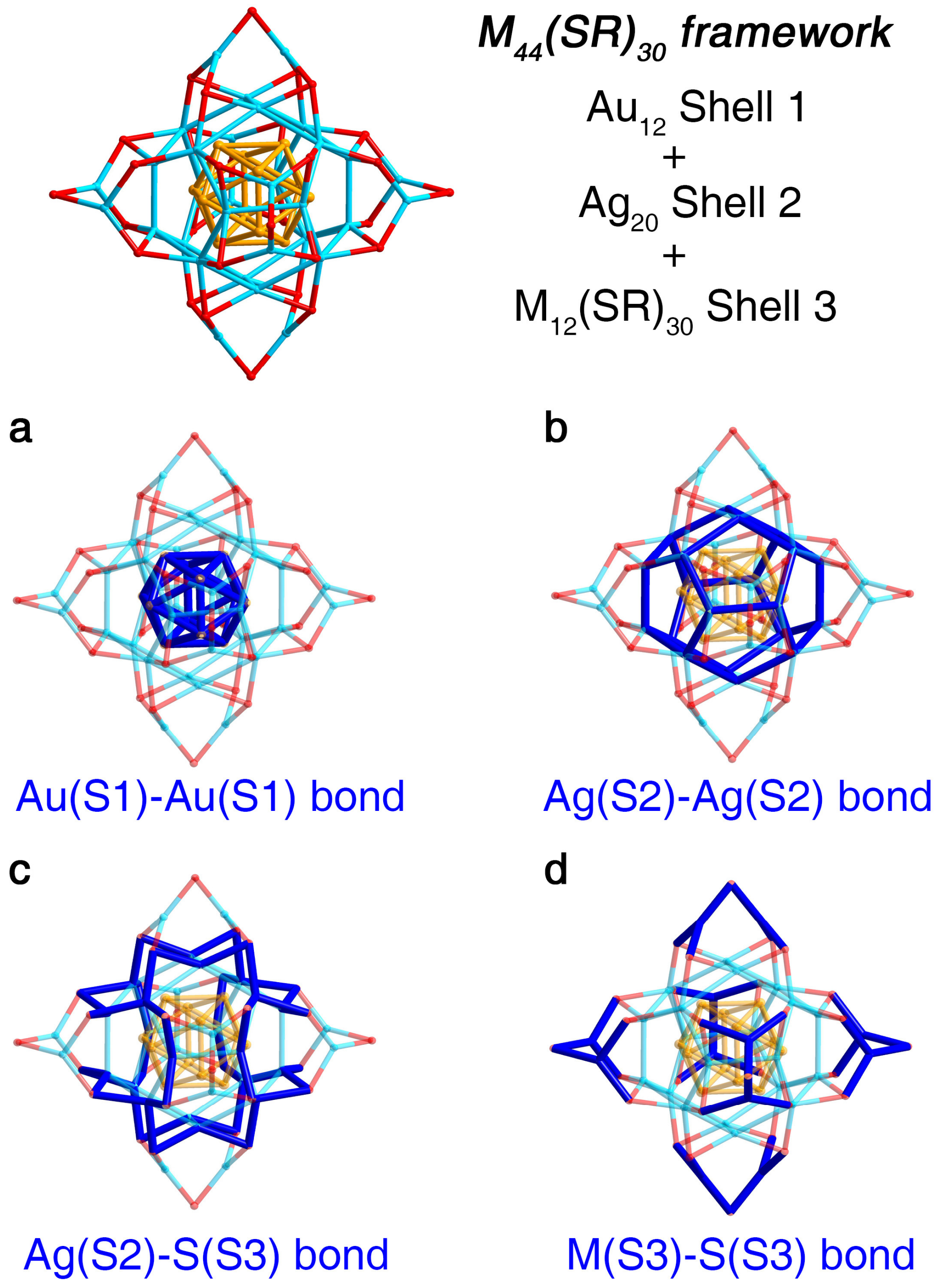

Supplementary Fig. 6 Comparison of the bond lengths (corresponding to Supplementary Table 2) between different $M_{44}(SR)_{30}$ (M = Au/Ag/Cu; SR = $SPhCl_2$) nanoclusters. **a** Au(S1)-Au(S1) bonds. **b** Ag(S2)-Ag(S2) bonds. **c** Ag(S2)-S(S3) bonds. **d** M(S3)-S(S3) bonds (M = Ag/Cu). The compared bonds are highlighted in blue.

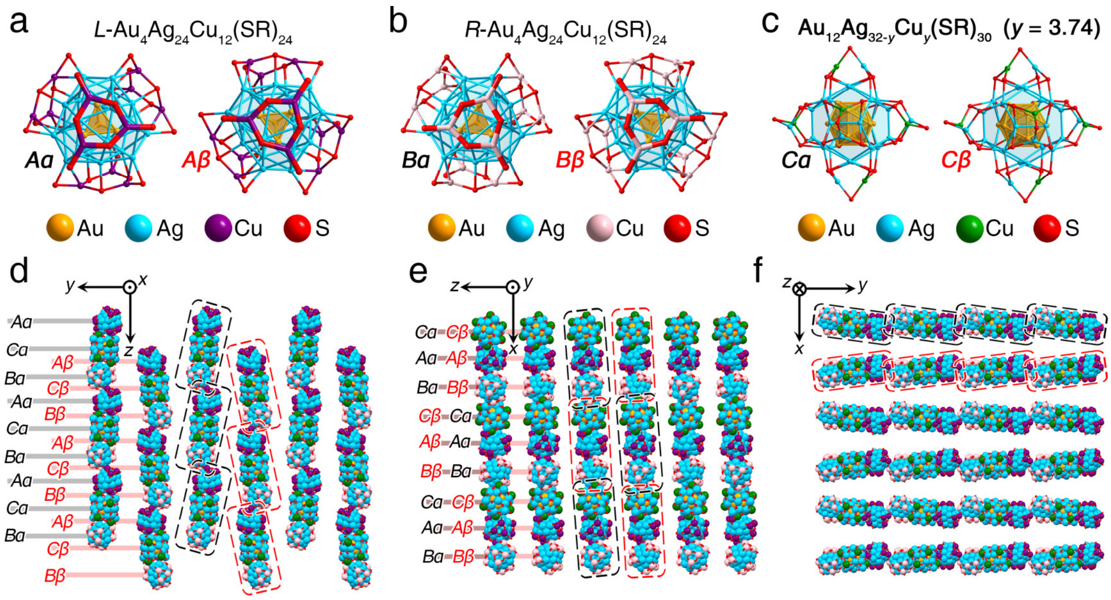

Supplementary Fig. 7 Co-crystallization of $Au_4Ag_{24}Cu_{12}(SR)_{24}$ and $Au_{12}Cu_yAg_{32-y}(SPhCl_2)_{30}$ (y = 3.74) nanoclusters. Structure of **a** *L*-$Au_4Ag_{24}Cu_{12}(SR)_{24}$, **b** *R*-$Au_4Ag_{24}Cu_{12}(SR)_{24}$, and **c** $Au_{12}Cu_yAg_{32-y}(SPhCl_2)_{30}$ (y = 3.74). Aα and Aβ (or Bα and Bβ, or Cα and Cβ) possess the same configuration, but in different angles. **d-f** Packing of the co-crystallized nanoclusters in the crystal lattice: view from the *x*-axis **d**, *y*-axis **e**, and *z*-axis **f**. The cluster units (Aα#Cα#Bα or Aβ#Cβ#Bβ) in red/black frames are the same.

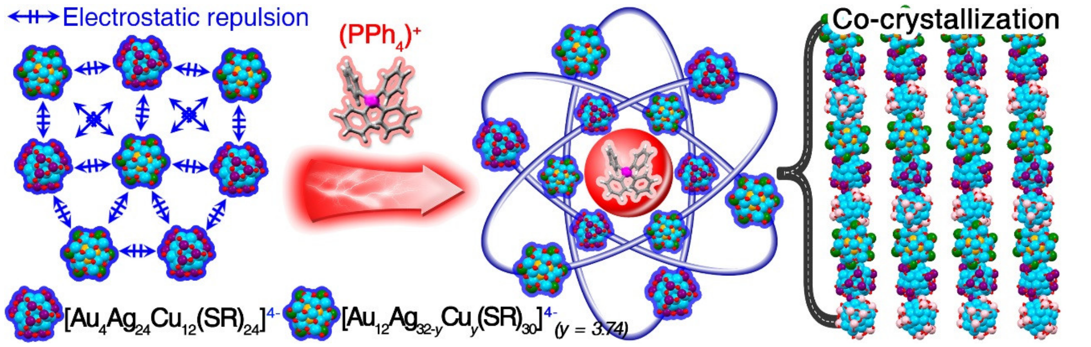

Supplementary Fig. 8 Illustration of the co-crystallization between two strongly negative valence nanoclusters induced by the presence of $(PPh_4)^+$ cations. The presence of $(PPh_4)^+$ reduces the electrostatic repulsion between adjacent $[Au_4Ag_{24}Cu_{12}(SR)_{24}]^{4-}$ and $[Au_{12}Cu_yAg_{32-y}(SPhCl_2)_{30}]^{4-}$ (y = 3.74) nanoclusters. Of note, the compositions of the co-crystallized nanoclusters may alter in the co-crystallization; indeed, the composition of $M_{40}(SR)_{24}$ nanocluster is determined as $Au_4Ag_{24}Cu_{12}(SR)_{24}$ although the pre-crystallized composition is $Au_xAg_{28-x}Cu_{12}(SR)_{24}$ where x ranges from 1-5 (corresponding to Stage 2 in Supplementary Fig. 14).

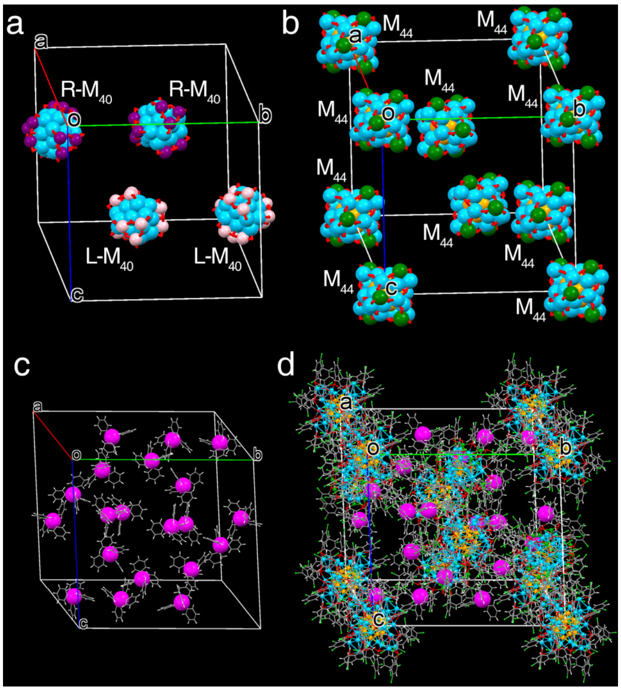

Supplementary Fig. 9 Packing of co-crystallized nanoclusters and counter-ions in a crystal lattice. **a** Packing of *R*-$Au_4Ag_{24}Cu_{12}(SR)_{24}$ and *L*-$Au_4Ag_{24}Cu_{12}(SR)_{24}$ nanoclusters in a crystal lattice. **b** Packing of $Au_{12}Cu_yAg_{32-y}(SPhCl_2)_{30}$ (y = 3.74) nanoclusters in a crystal lattice. **c** Packing of $(PPh_4)^+$ cations in a crystal lattice. **d** The overall structure of a crystal lattice. Color legends: orange sphere, Au; blue sphere, Ag; purple/pink/green sphere, Cu in different nanoclusters; red sphere, S. For clarity, some H and C atoms are omitted.

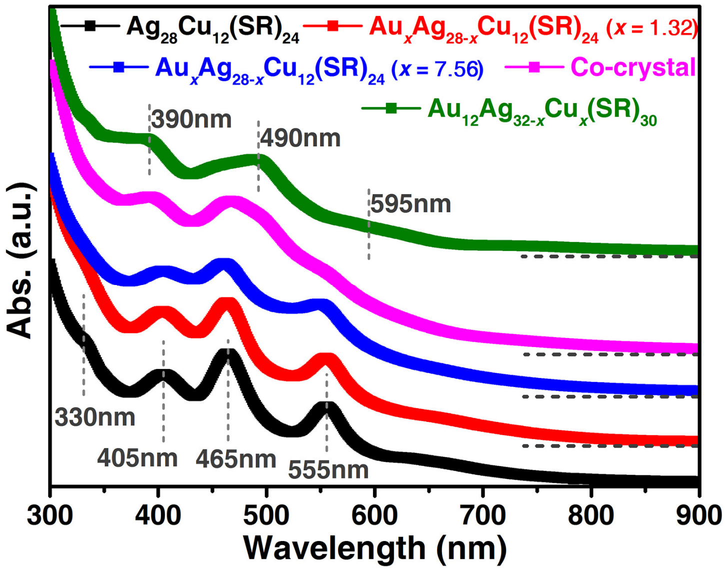

Supplementary Fig. 10 Optical absorptions of different M_{40} or M_{44} nanoclusters. The optical absorption of $Ag_{28}Cu_{12}(SPhCl_2)_{24}$ is in black. The optical absorption of $Au_xAg_{28-x}Cu_{12}(SPhCl_2)_{24}$ (x = 1.32) is in red. The optical absorption of $Au_xAg_{28-x}Cu_{12}(SPhCl_2)_{24}$ (x = 7.56) is in blue. The optical absorption of the co-crystallized $Au_4Ag_{24}Cu_{12}(SPhCl_2)_{24}$ and $Au_{12}Cu_yAg_{32-y}(SPhCl_2)_{30}$ (y = 3.74) is in magenta. The optical absorption of $Au_{12}Ag_{32-x}Cu_x(SPhCl_2)_{30}$ is in green. a.u., arbitrary units.

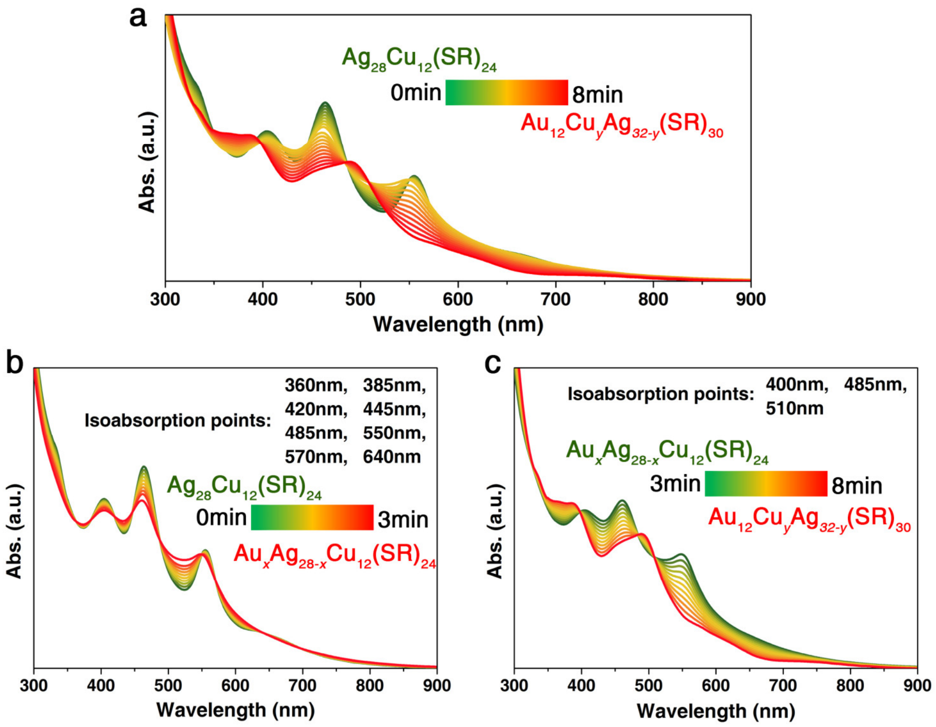

Supplementary Fig. 11 Time-dependent UV-vis spectra of the transformation from $[Ag_{28}Cu_{12}(SPhCl_2)_{24}]^{4-}$ to $[Au_xAg_{28\text{-}x}Cu_{12}(SPhCl_2)_{24}]^{4-}$ and then to $[Au_{12}Cu_yAg_{32\text{-}y}(SPhCl_2)_{30}]^{4-}$. **a** Detection from 0 to 7 min, corresponding to the transformation from $[Ag_{28}Cu_{12}(SPhCl_2)_{24}]^{4-}$ to $[Au_{12}Cu_yAg_{32\text{-}y}(SPhCl_2)_{30}]^{4-}$. **b** Detection from 0 to 3 min, corresponding to the transformation from $[Ag_{28}Cu_{12}(SPhCl_2)_{24}]^{4-}$ to $[Au_xAg_{28\text{-}x}Cu_{12}(SPhCl_2)_{24}]^{4-}$. **c** Detection from 3 to 8 min, corresponding to the transformation from $[Au_xAg_{28\text{-}x}Cu_{12}(SPhCl_2)_{24}]^{4-}$ to $[Au_{12}Cu_yAg_{32\text{-}y}(SPhCl_2)_{30}]^{4-}$. a.u., arbitrary units.

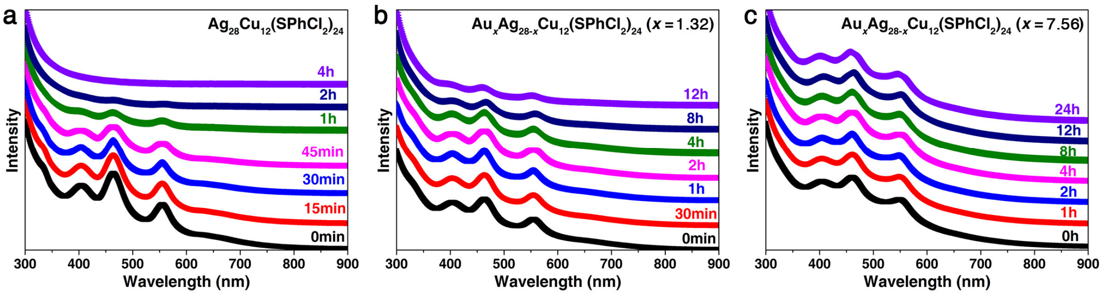

Supplementary Fig. 12 Time-dependent UV-vis spectra of different $M_{40}(SR)_{24}$ nanoclusters to test the thermal stability of the nanocluster. All nanocluster samples were dissolved in CH_2Cl_2 and kept at 35°C. **a** Time-dependent UV-vis spectra of $Ag_{28}Cu_{12}(SPhCl_2)_{24}$. **b** Time-dependent UV-vis spectra of $Au_xAg_{28\text{-}x}Cu_{12}(SPhCl_2)_{24}$ (x = 1.32). **c** Time-dependent UV-vis spectra of $Au_xAg_{28\text{-}x}Cu_{12}(SPhCl_2)_{24}$ (x = 7.56).

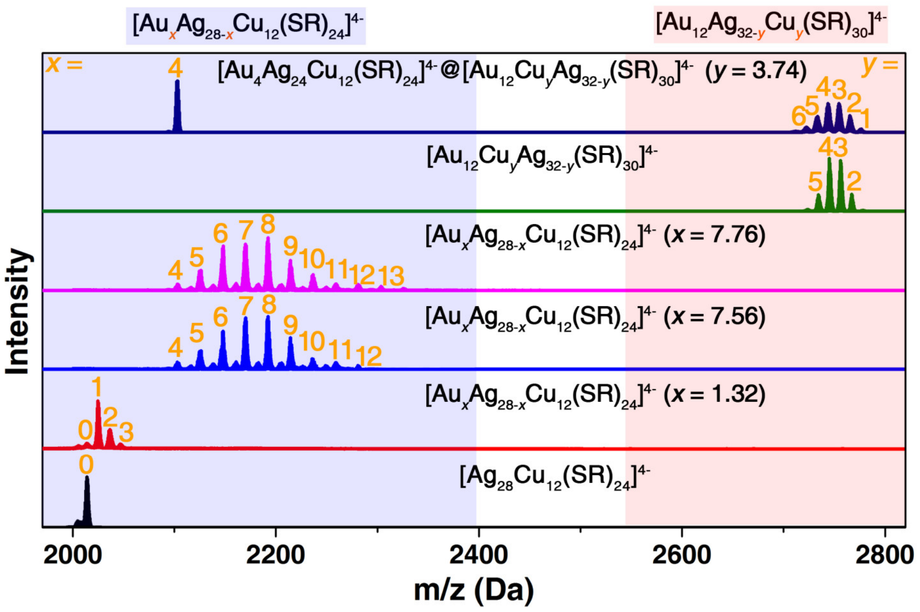

Supplementary Fig. 13 ESI-MS results of different nanocluster crystals, including $[Ag_{28}Cu_{12}(SR)_{24}]^{4-}$, $[Au_xAg_{28-x}Cu_{12}(SR)_{24}]^{4-}$ (x = 1.32), $[Au_xAg_{28-x}Cu_{12}(SR)_{24}]^{4-}$ (x = 7.56), $[Au_xAg_{28-x}Cu_{12}(SR)_{24}]^{4-}$ (x = 7.76), $[Au_{12}Cu_yAg_{32-y}(SR)_{30}]^{4-}$, and $[Au_4Ag_{24}Cu_{12}(SR)_{24}]^{4-}{}_2$@$[Au_{12}Cu_yAg_{32-y}(SR)_{30}]^{4-}{}_1$ (y = 3.74). All cluster crystals were dissolved in CH_2Cl_2. For the MASS result of $[Au_xAg_{28-x}Cu_{12}(Sr)_{24}]^{4-}$ (x = 1.32), a total of four mass signals were detected, $[Au_xAg_{28-x}Cu_{12}(SPhCl_2)_{24}]^{4-}$ (x = 0, 1, 2, 3). In this context, the crystal structure of $[Au_xAg_{28-x}Cu_{12}(SPhCl_2)_{24}]^{4-}$ (x = 1.32) should represent the co-crystallization of $\{[Ag_{28}Cu_{12}(SR)_{24}]_a$@$[Au_1Ag_{27}Cu_{12}(SR)_{24}]_b$@$[Au_2Ag_{26}Cu_{12}(SR)_{24}]_c$@$[Au_3Ag_{25}Cu_{12}(SR)_{24}]_d\}$, and the $[Au_{1.32}Ag_{26.68}Cu_{12}(SPhCl_2)_{24}]^{4-}$ was a homogenized result (the same for other cases).

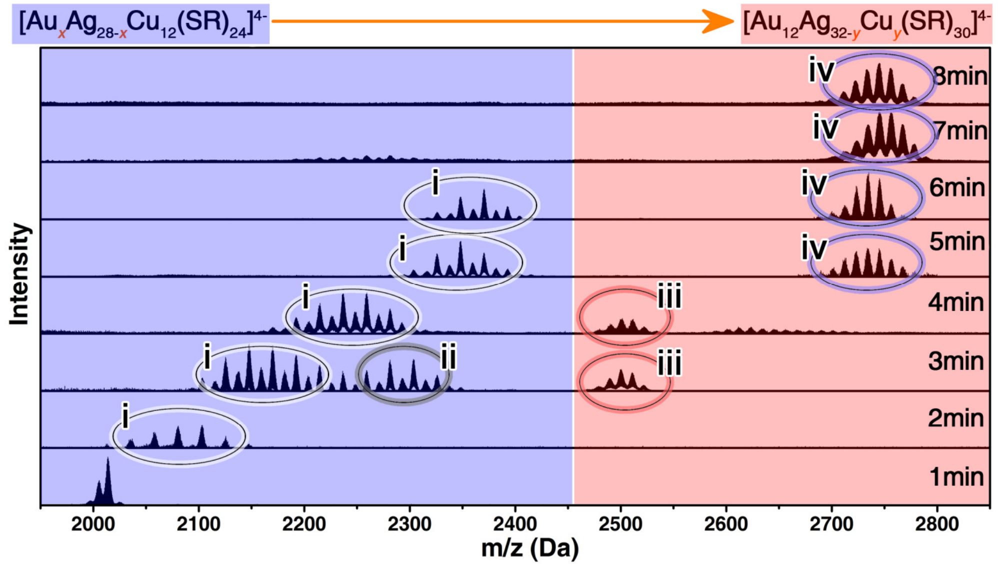

Supplementary Fig. 14 Time-dependent ESI-MS results of Au alloying-induced transformation from $[M_{40}(SR)_{24}]^{4-}$ to $[M_{44}(SR)_{30}]^{4-}$ nanoclusters with a range from 1950 to 2850 Da. The signals with blue backgrounds represent $[Au_xAg_{28-x}Cu_{12}(SR)_{24}]^{4-}$ with different x values. The signals with red backgrounds represent $[Au_{12}Ag_{32-y}Cu_y(SR)_{30}]^{4-}$ with different y values.

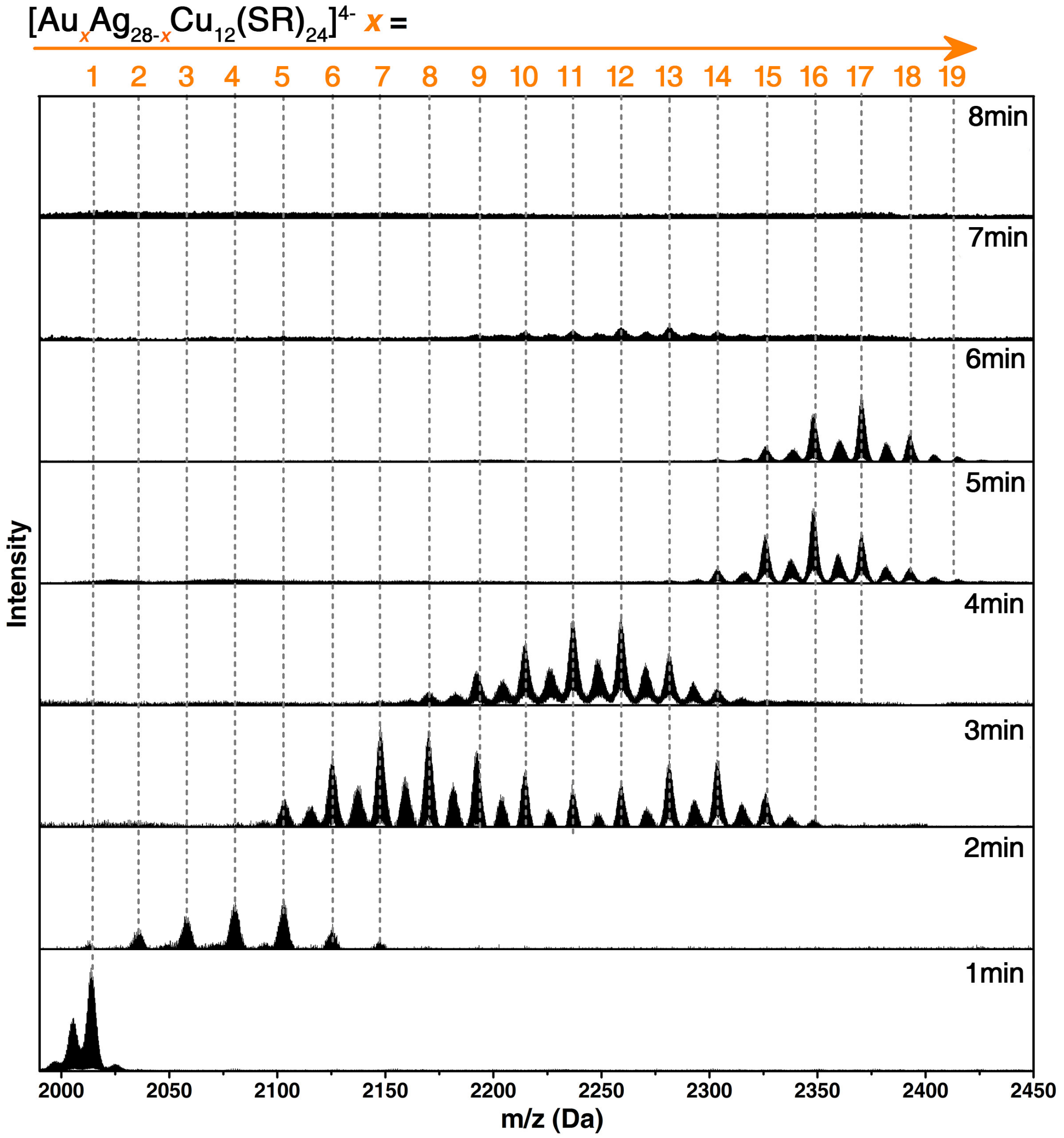

Supplementary Fig. 15 Time-dependent ESI-MS results of Au alloying process from $[Ag_{28}Cu_{12}(SPhCl_2)_{24}]^{4-}$ with a range from 1950 to 2450 Da. Each marked peak in the figure represents the signal of $[Au_xAg_{28-x}Cu_{12}(SR)_{24}]^{4-}$ with different x values.

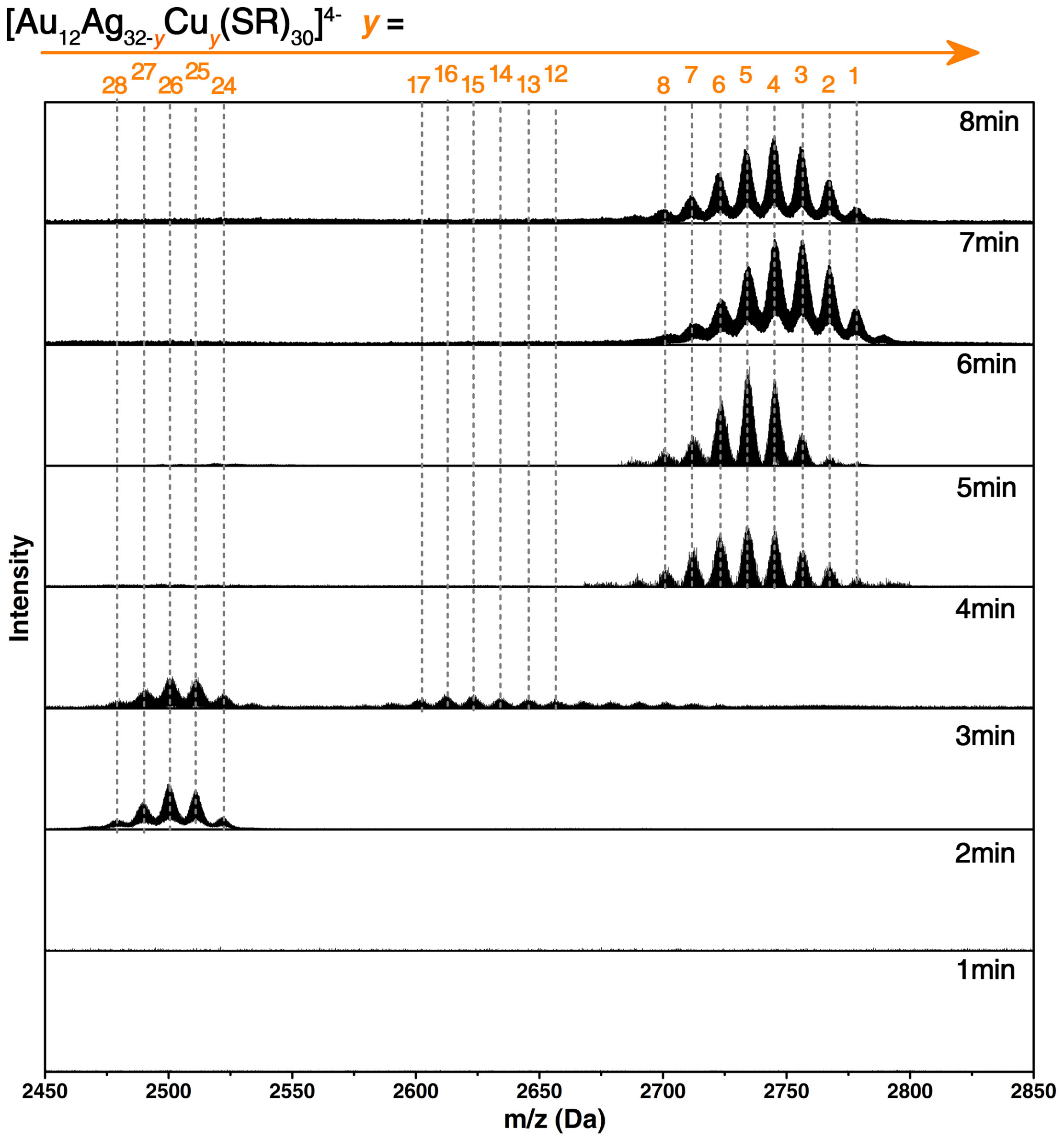

Supplementary Fig. 16 Time-dependent ESI-MS results of Au alloying process for the generation of $[M_{44}(SR)_{30}]^{4-}$ nanoclusters with a range from 2450 to 2850 Da. Each marked peak in the figure represents the signal of $[Au_{12}Ag_{32-y}Cu_y(SPhCl_2)_{30}]^{4-}$ with different *y* values.

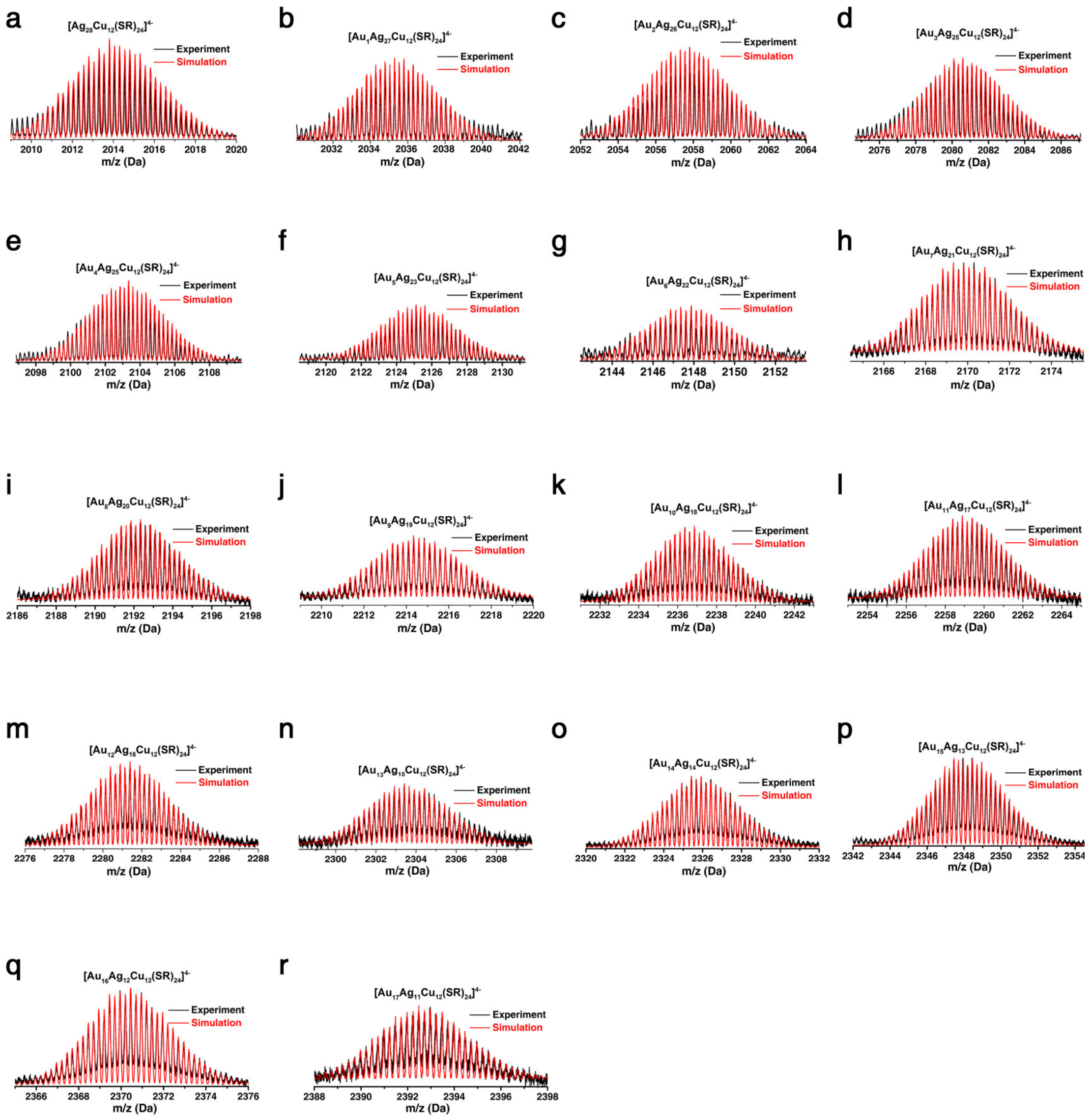

Supplementary Fig. 17 Experimental (in black) and calculated (in red) isotope patterns of **a** $[Ag_{28}Cu_{12}(SPhCl_2)_{24}]^{4-}$, **b** $[Au_1Ag_{27}Cu_{12}(SPhCl_2)_{24}]^{4-}$, **c** $[Au_2Ag_{26}Cu_{12}(SPhCl_2)_{24}]^{4-}$, **d** $[Au_3Ag_{25}Cu_{12}(SPhCl_2)_{24}]^{4-}$, **e** $[Au_4Ag_{24}Cu_{12}(SPhCl_2)_{24}]^{4-}$, **f** $[Au_5Ag_{23}Cu_{12}(SPhCl_2)_{24}]^{4-}$, **g** $[Au_6Ag_{22}Cu_{12}(SPhCl_2)_{24}]^{4-}$, **h** $[Au_7Ag_{21}Cu_{12}(SPhCl_2)_{24}]^{4-}$, **i** $[Au_8Ag_{20}Cu_{12}(SPhCl_2)_{24}]^{4-}$, **j** $[Au_9Ag_{19}Cu_{12}(SPhCl_2)_{24}]^{4-}$, **k** $[Au_{10}Ag_{18}Cu_{12}(SPhCl_2)_{24}]^{4-}$, **l** $[Au_{11}Ag_{17}Cu_{12}(SPhCl_2)_{24}]^{4-}$, **m** $[Au_{12}Ag_{16}Cu_{12}(SPhCl_2)_{24}]^{4-}$, **n** $[Au_{13}Ag_{15}Cu_{12}(SPhCl_2)_{24}]^{4-}$, **o** $[Au_{14}Ag_{14}Cu_{12}(SPhCl_2)_{24}]^{4-}$, **p** $[Au_{15}Ag_{13}Cu_{12}(SPhCl_2)_{24}]^{4-}$, **q** $[Au_{16}Ag_{12}Cu_{12}(SPhCl_2)_{24}]^{4-}$, and **r** $[Au_{17}Ag_{11}Cu_{12}(SPhCl_2)_{24}]^{4-}$ nanoclusters.

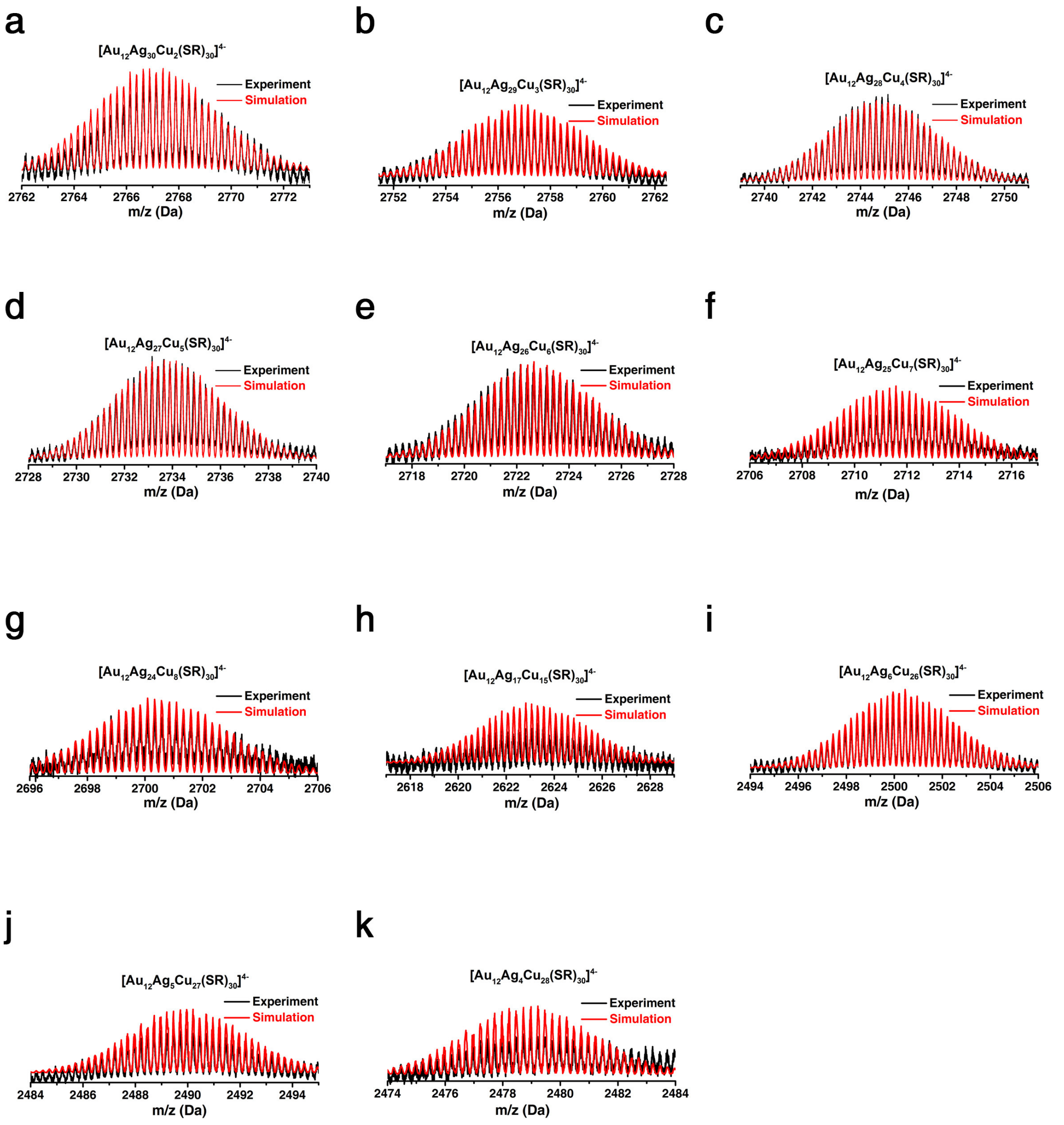

Supplementary Fig. 18 Experimental (in black) and calculated (in red) isotope patterns of **a** $[Au_{12}Ag_{30}Cu_2(SPhCl_2)_{30}]^{4-}$, **b** $[Au_{12}Ag_{29}Cu_3(SPhCl_2)_{30}]^{4-}$, **c** $[Au_{12}Ag_{28}Cu_4(SPhCl_2)_{30}]^{4-}$, **d** $[Au_{12}Ag_{27}Cu_5(SPhCl_2)_{30}]^{4-}$, **e** $[Au_{12}Ag_{26}Cu_6(SPhCl_2)_{30}]^{4-}$, **f** $[Au_{12}Ag_{25}Cu_7(SPhCl_2)_{30}]^{4-}$, **g** $[Au_{12}Ag_{24}Cu_8(SPhCl_2)_{30}]^{4-}$, **h** $[Au_{12}Ag_{17}Cu_{15}(SPhCl_2)_{30}]^{4-}$, **i** $[Au_{12}Ag_6Cu_{26}(SPhCl_2)_{30}]^{4-}$, **j** $[Au_{12}Ag_5Cu_{27}(SPhCl_2)_{30}]^{4-}$, and **k** $[Au_{12}Ag_4Cu_{28}(SPhCl_2)_{30}]^{4-}$ nanoclusters.

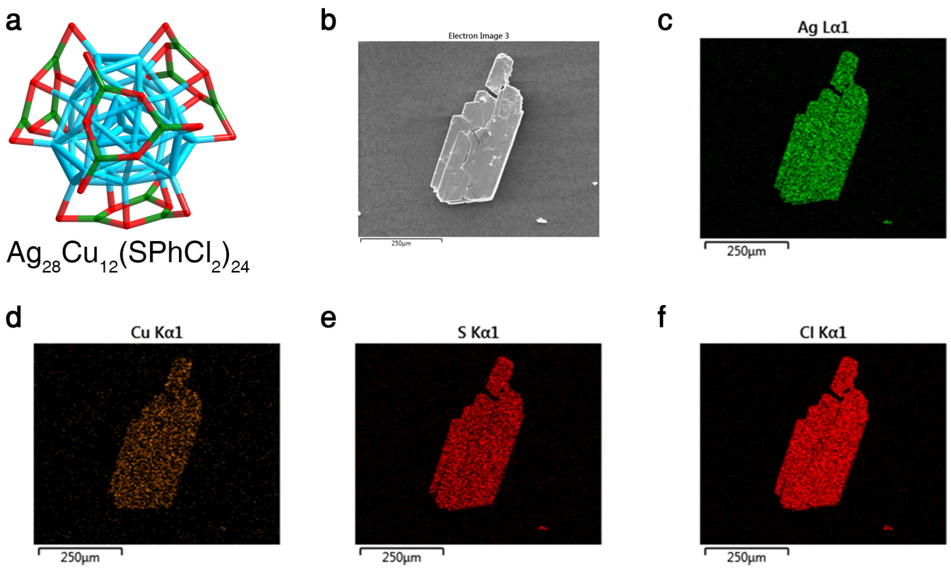

Supplementary Fig. 19 a Crystal structure of $Ag_{28}Cu_{12}(SPhCl_2)_{24}$. **b-f** Elemental mapping images of the $Ag_{28}Cu_{12}(SPhCl_2)_{24}$ nanocluster crystal.

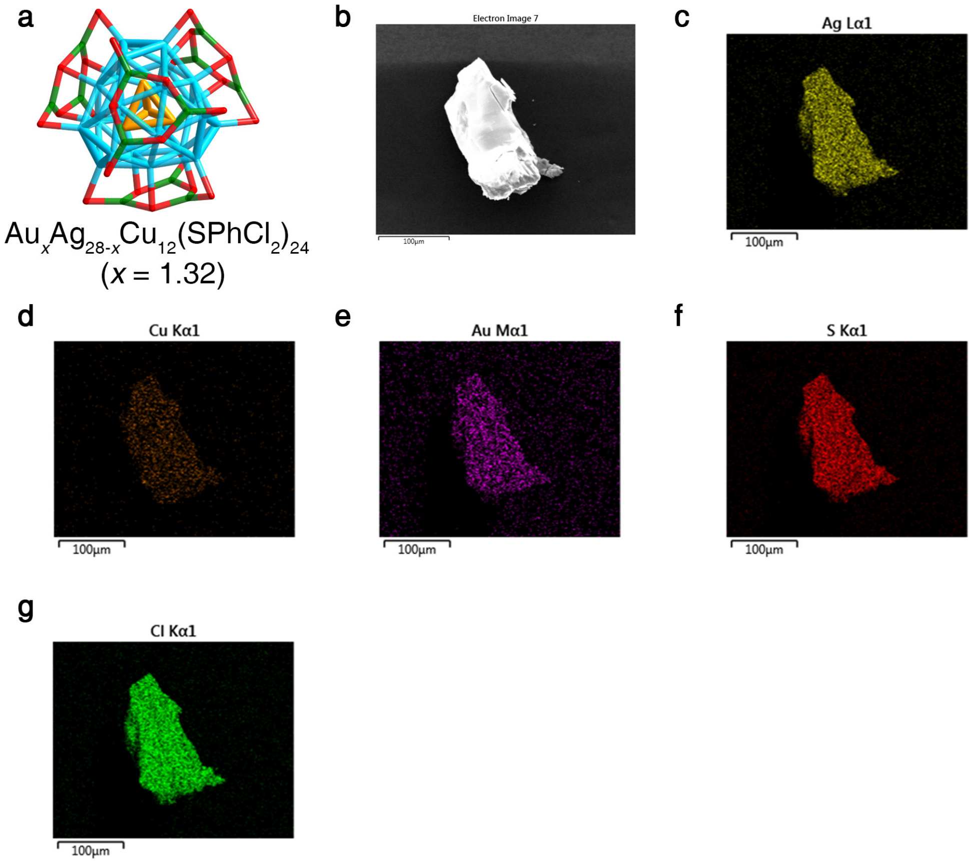

Supplementary Fig. 20 a Crystal structure of $Au_xAg_{28-x}Cu_{12}(SPhCl_2)_{24}$ (x = 1.32). **b-g** Elemental mapping images of the $Au_xAg_{28-x}Cu_{12}(SPhCl_2)_{24}$ (x = 1.32) nanocluster crystal.

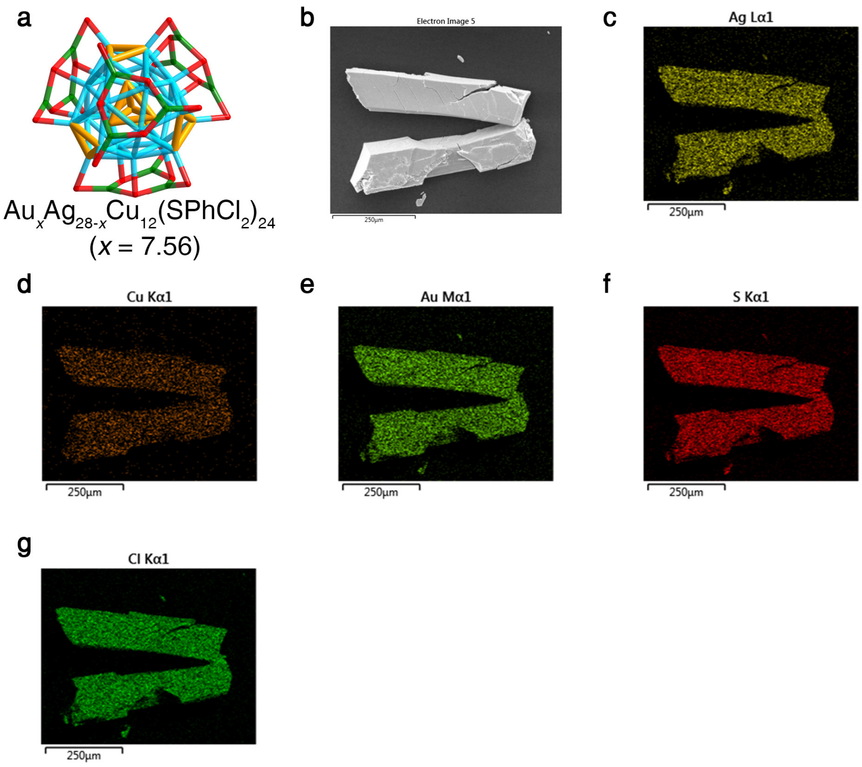

Supplementary Fig. 21 **a** Crystal structure of $Au_xAg_{28-x}Cu_{12}(SPhCl_2)_{24}$ (x = 7.56). **b-g** Elemental mapping images of the $Au_xAg_{28-x}Cu_{12}(SPhCl_2)_{24}$ (x = 7.56) nanocluster crystal.

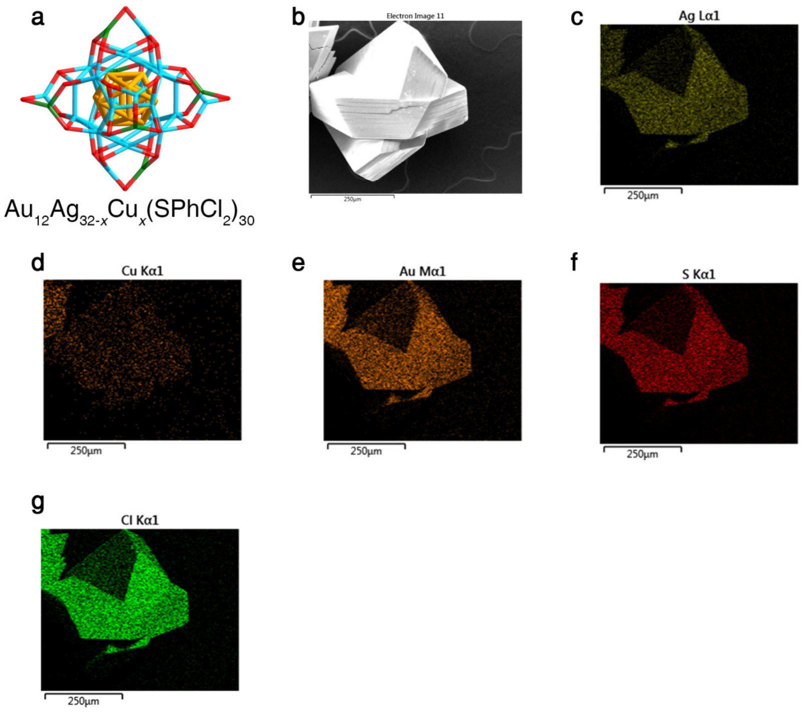

Supplementary Fig. 22 **a** Crystal structure of $Au_{12}Ag_{32-x}Cu_x(SPhCl_2)_{30}$. **b-g** Elemental mapping images of the $Au_{12}Ag_{32-x}Cu_x(SPhCl_2)_{30}$ nanocluster crystal.

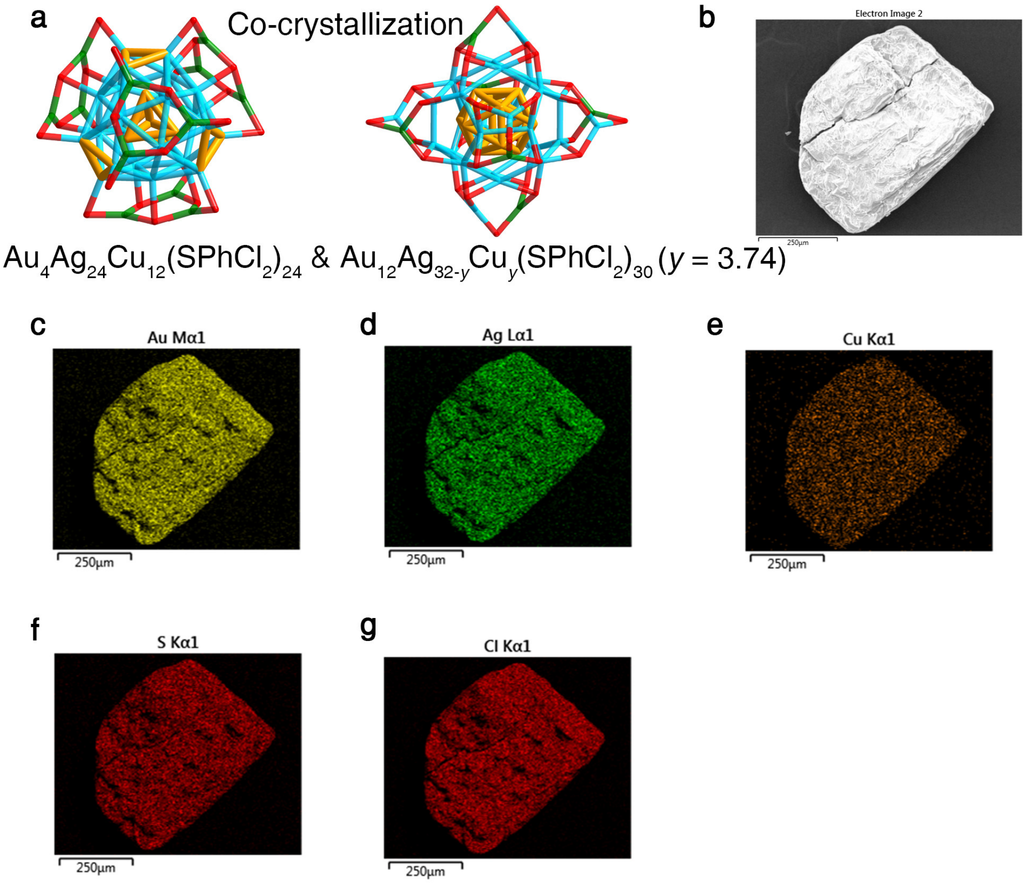

Supplementary Fig. 23 **a** Crystal structures of the co-crystallized $Au_4Ag_{24}Cu_{12}(SPhCl_2)_{24}$ and $Au_{12}Cu_yAg_{32-y}(SPhCl_2)_{30}$ (y = 3.74) nanoclusters. **b-g** Elemental mapping images of the co-crystallized $Au_4Ag_{24}Cu_{12}(SPhCl_2)_{24}$ and $Au_{12}Cu_yAg_{32-y}(SPhCl_2)_{30}$ (y = 3.74) nanoclusters crystal.

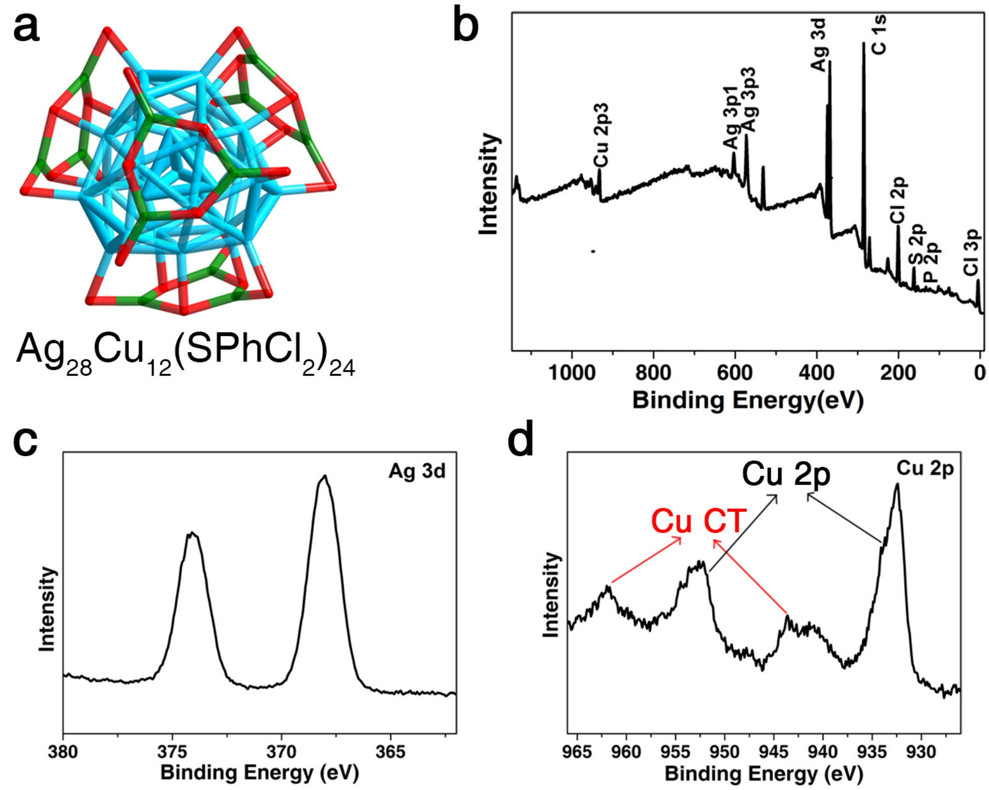

Supplementary Fig. 24 **a** Crystal structure of $Ag_{28}Cu_{12}(SPhCl_2)_{24}$. **b** XPS spectrum of the $Ag_{28}Cu_{12}(SPhCl_2)_{24}$ nanoclusters. **c** XPS of Ag 3d of the $Ag_{28}Cu_{12}(SPhCl_2)_{24}$ nanocluster. **d** XPS of Cu 2p of the $Ag_{28}Cu_{12}(SPhCl_2)_{24}$ nanocluster. The Cu 2p peaks (932.6 and 952.2 eV) were the same as those peaks observed in other cluster samples (Supplementary Figs. 25-28), demonstrating the similar charge states of these Cu atoms (+1) in each nanocluster. By comparison, the 942.2 and 962.5 eV represented the Cu CT (charge transfer) bands, which were also observed in the sample of co-crystallized $Au_4Ag_{24}Cu_{12}(SPhCl_2)_{24}$ and $Au_{12}Cu_yAg_{32-y}(SPhCl_2)_{30}$ (Supplementary Fig. 28). The Cu CT band was not the contribution from the general s, d, and p orbital electrons from the nanocluster molecule, but the contribution from the electron transfer between different atoms within the nanocluster or between different molecules (Phys. Rev. B, 2002, 66, 045105). In this context, these two cluster samples (i.e., $Ag_{28}Cu_{12}(SPhCl_2)_{24}$ and co-crystallized $Au_4Ag_{24}Cu_{12}(SPhCl_2)_{24}$#$Au_{12}Cu_yAg_{32-y}(SPhCl_2)_{30}$) might display different intercluster interactions relative to the other cluster samples (i.e., $Au_xAg_{28-x}Cu_{12}(SPhCl_2)_{24}$ (x = 1.32), $Au_xAg_{28-x}Cu_{12}(SPhCl_2)_{24}$ (x = 7.56), and $Au_{12}Ag_{32-x}Cu_x(SPhCl_2)_{30}$).

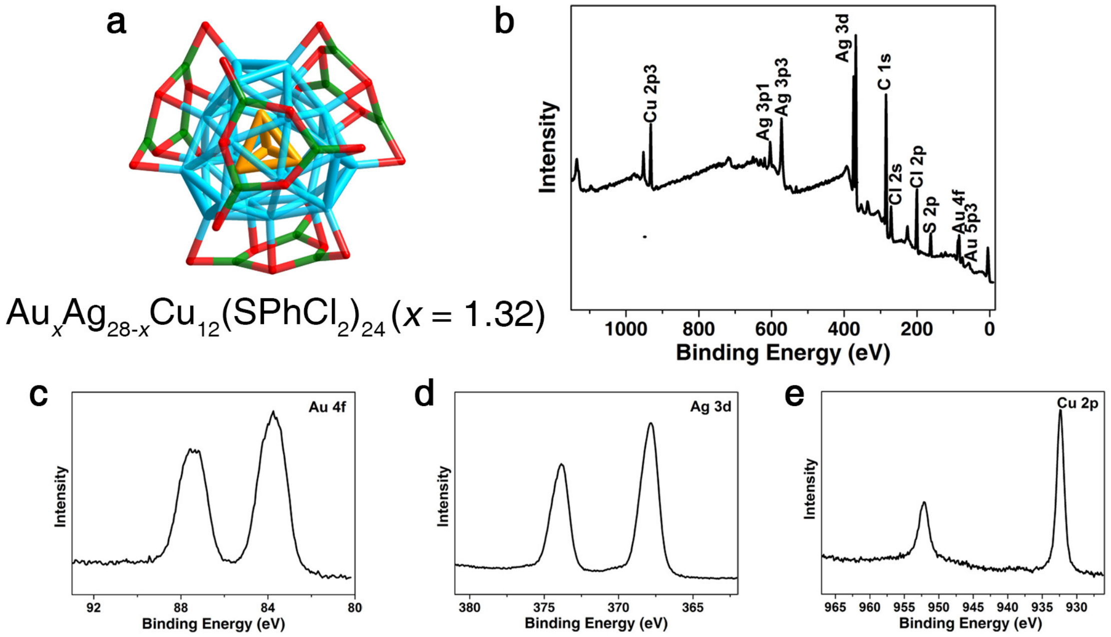

Supplementary Fig. 25 a Crystal structure of $Au_xAg_{28-x}Cu_{12}(SPhCl_2)_{24}$ (x = 1.32). **b** XPS spectrum of the $Au_xAg_{28-x}Cu_{12}(SPhCl_2)_{24}$ (x = 1.32) nanoclusters. **c** XPS of Au 4f of the $Au_xAg_{28-x}Cu_{12}(SPhCl_2)_{24}$ (x = 1.32) nanocluster. **d** XPS of Ag 3d of the $Au_xAg_{28-x}Cu_{12}(SPhCl_2)_{24}$ (x = 1.32) nanocluster. **e** XPS of Cu 2p of the $Au_xAg_{28-x}Cu_{12}(SPhCl_2)_{24}$ (x = 1.32) nanocluster.

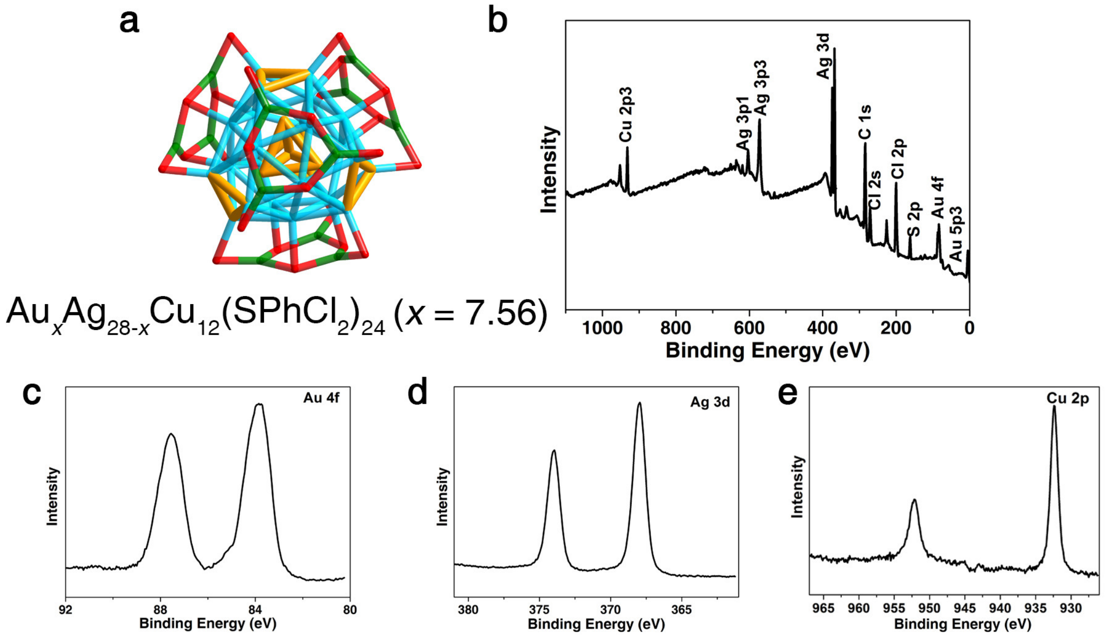

Supplementary Fig. 26 a Crystal structure of $Au_xAg_{28-x}Cu_{12}(SPhCl_2)_{24}$ (x = 7.56). **b** XPS spectrum of the $Au_xAg_{28-x}Cu_{12}(SPhCl_2)_{24}$ (x = 7.56) nanoclusters. **c** XPS of Au 4f of the $Au_xAg_{28-x}Cu_{12}(SPhCl_2)_{24}$ (x = 7.56) nanocluster. **d** XPS of Ag 3d of the $Au_xAg_{28-x}Cu_{12}(SPhCl_2)_{24}$ (x = 7.56) nanocluster. **e** XPS of Cu 2p of the $Au_xAg_{28-x}Cu_{12}(SPhCl_2)_{24}$ (x = 7.56) nanocluster.

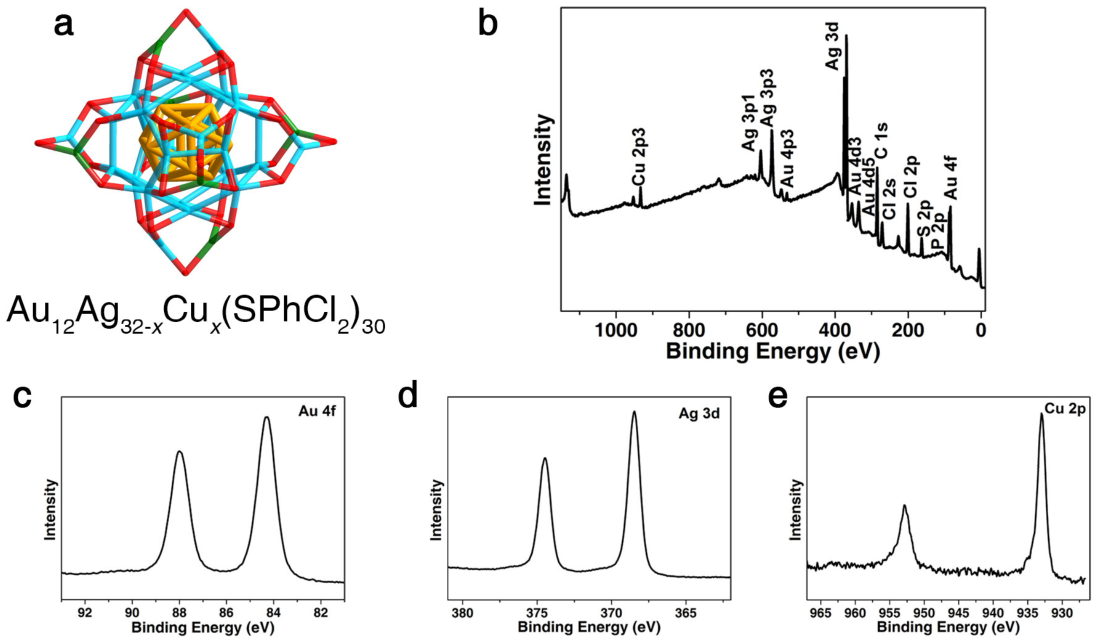

Supplementary Fig. 27 **a** Crystal structure of $Au_{12}Ag_{32\text{-}x}Cu_x(SPhCl_2)_{30}$. **b** XPS spectrum of the $Au_{12}Ag_{32\text{-}x}Cu_x(SPhCl_2)_{30}$ nanoclusters. **c** XPS of Au 4f of the $Au_{12}Ag_{32\text{-}x}Cu_x(SPhCl_2)_{30}$ nanocluster. **d** XPS of Ag 3d of the $Au_{12}Ag_{32\text{-}x}Cu_x(SPhCl_2)_{30}$ nanocluster. **e** XPS of Cu 2p of the $Au_{12}Ag_{32\text{-}x}Cu_x(SPhCl_2)_{30}$ nanocluster.

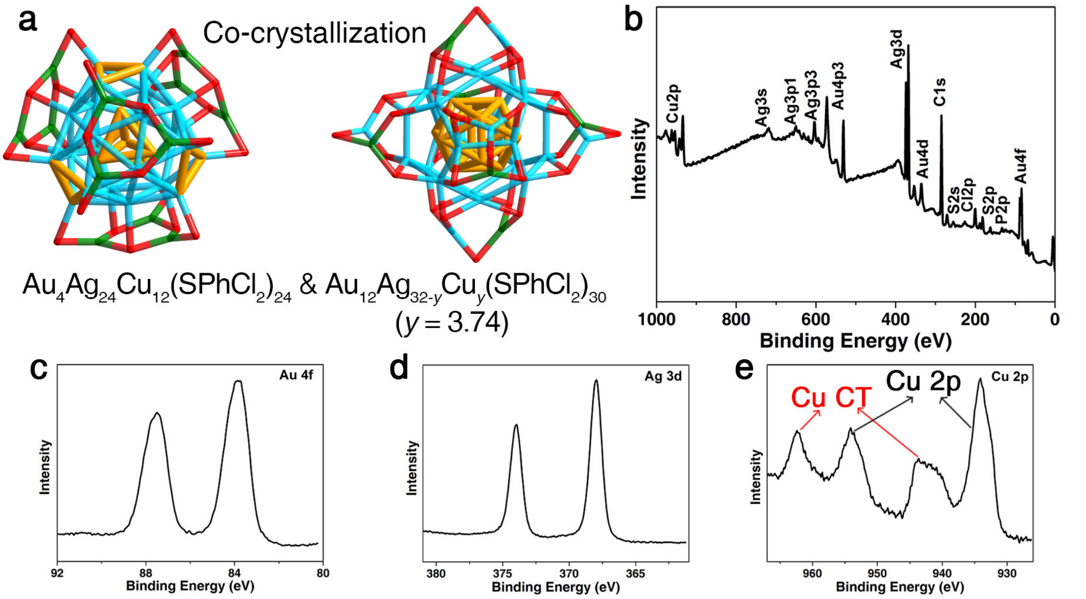

Supplementary Fig. 28 **a** Crystal structure of the co-crystallized $Au_4Ag_{24}Cu_{12}(SPhCl_2)_{24}$ and $Au_{12}Cu_yAg_{32\text{-}y}(SPhCl_2)_{30}$ (y = 3.74) nanoclusters. **b** XPS spectrum of the co-crystallized $Au_4Ag_{24}Cu_{12}(SPhCl_2)_{24}$ and $Au_{12}Cu_yAg_{32\text{-}y}(SPhCl_2)_{30}$ (y = 3.74) nanoclusters. **c** XPS of Au 4f of the co-crystallized $Au_4Ag_{24}Cu_{12}(SPhCl_2)_{24}$ and $Au_{12}Cu_yAg_{32\text{-}y}(SPhCl_2)_{30}$ (y = 3.74) nanoclusters. **d** XPS of Ag 3d of the co-crystallized $Au_4Ag_{24}Cu_{12}(SPhCl_2)_{24}$ and $Au_{12}Cu_yAg_{32\text{-}y}(SPhCl_2)_{30}$ (y = 3.74) nanoclusters. **e** XPS of Cu 2p of the co-crystallized $Au_4Ag_{24}Cu_{12}(SPhCl_2)_{24}$ and $Au_{12}Cu_yAg_{32\text{-}y}(SPhCl_2)_{30}$ (y = 3.74) nanoclusters.

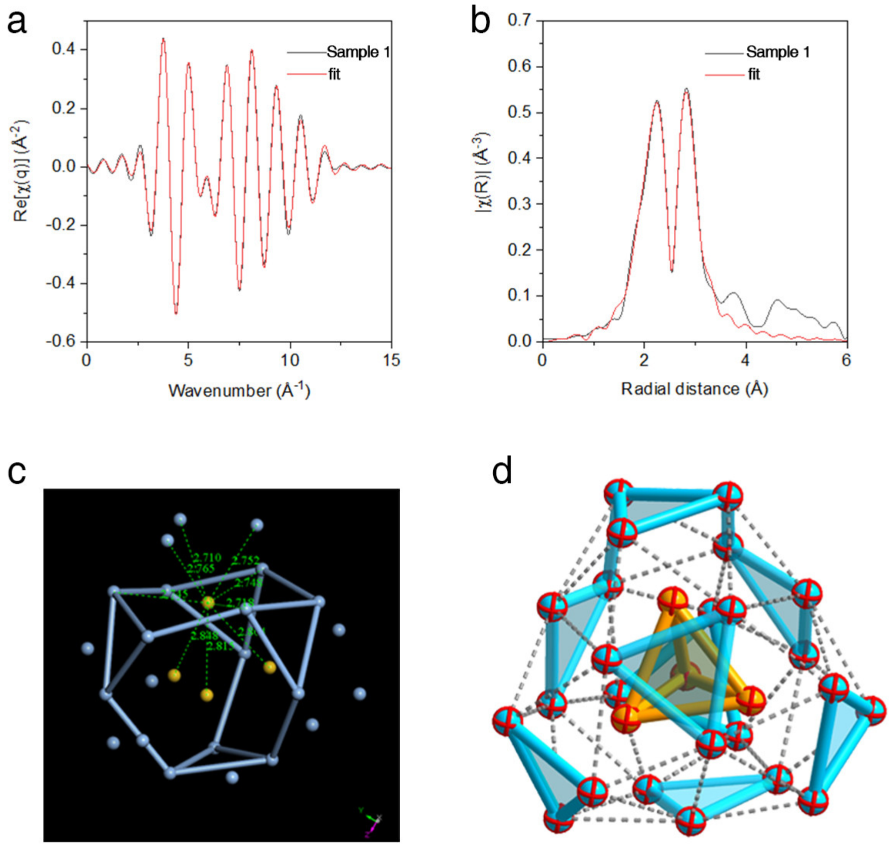

Supplementary Fig. 29 **a** The fitting curve of $k^2\chi(k)$ oscillations and **b** corresponding Fourier transforms for Sample 1 corresponding to Supplementary Table 3. **c** Simulated Au doping positions derive from EXAFS results. **d** The matching kernel structure of Sample 1 derive from EXAFS results. In this M_{40} nanocluster sample, the introduced Au heteroatoms only occupied the innermost tetrahedral kernel. As shown in the Au L_3-edge EXAFS spectrum of sample 1, the peak position at ~2.3 Å is significantly higher than the ~1.9 Å of Au-S coordination peak (Supplementary Fig. 30). Therefore, this peak cannot be ascribed to Au-S, but corresponds to Au-M (M = metal). It confirms that no Au-S band exists in sample 1, and Au atoms are located in the M_4 tetrahedron. The split of two peaks can be attributed to the nonlinear effect from heavy 6th-period metal (a similar phenomenon can be found in the EXAFS spectrum of Au or Pt foil), and the contribution from two bonds of Au-Au and Au-Ag (Table S4).

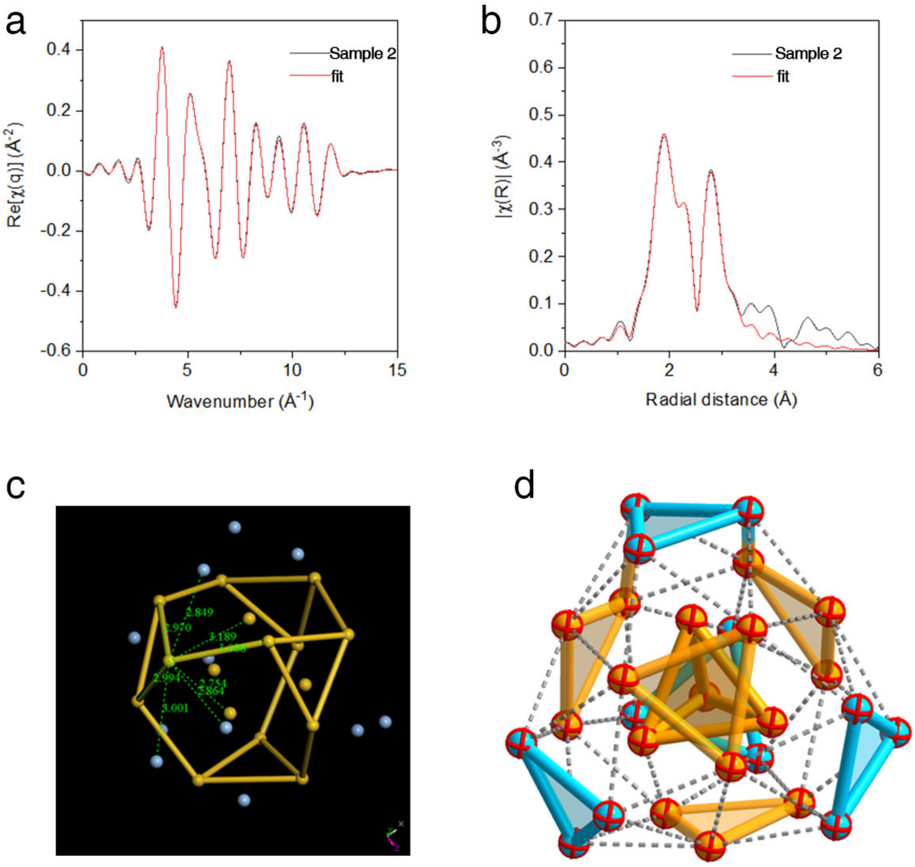

Supplementary Fig. 30 **a** The fitting curve of $k^2\chi(k)$ oscillations and **b** corresponding Fourier transforms for Sample 2 corresponding to Supplementary Table 4. **c** Simulated Au doping positions derive from EXAFS results. **d** The matching kernel structure of Sample 2 derive from EXAFS results. In this M_{40} nanocluster sample, the introduced Au heteroatoms not only occupied the innermost tetrahedral kernel, but also substituted the Ag atoms in *unstable locations*, different from the crystal result wherein the *unstable locations* were maintained as Ag throughout (Supplementary Fig. 3).

Supplementary Table 1 Comparison of the bond lengths (corresponding to Supplementary Fig. 5) among different $M_{40}(SR)_{24}$ nanoclusters.

Bond type	$Ag_{28}Cu_{12}$	$Au_xAg_{28-x}Cu_{12}$ (x = 1.32)	$Au_xAg_{28-x}Cu_{12}$ (x = 7.56)	$Au_4Ag_{24}Cu_{12}$ (Co-crystallization)
M(S1)-M(S1)	Range: 2.804 Å---2.840 Å<br>Avg. 2.824 Å	Range: 2.817 Å---2.850 Å<br>Avg. 2.830 Å	Range: 2.804 Å---2.854 Å<br>Avg. 2.828 Å	Range: 2.825 Å---2.854 Å<br>Avg. 2.836 Å
M(S2)-M(S2)	Range: 2.793 Å---3.081 Å<br>Avg. 2.942 Å	Range: 2.802 Å---3.072 Å<br>Avg. 2.939 Å	Range: 2.790 Å---3.159 Å<br>Avg. 2.943 Å	Range: 2.809 Å---3.084 Å<br>Avg. 2.936 Å
M(S2)-S(S3)	Range: 2.480 Å—2.635 Å<br>Avg. 2.549 Å	Range: 2.476 Å---2.607 Å<br>Avg. 2.540 Å	Range: 2.434 Å---2.609 Å<br>Avg. 2.518 Å	Range: 2.453 Å---2.615 Å<br>Avg. 2.531 Å
Cu(S3)-S(S3)	Range: 2.204 Å---2.321 Å<br>Avg. 2.265 Å	Range: 2.210 Å---2.293 Å<br>Avg. 2.254 Å	Range: 2.222 Å---2.292 Å<br>Avg. 2.252 Å	Range: 2.218 Å---2.289 Å<br>Avg. 2.246 Å

Supplementary Table 2 Comparison of the bond lengths (corresponding to Supplementary Fig. 6) between different $M_{44}(SR)_{30}$ nanoclusters.

Bond type	$Au_{12}Ag_{32}$	$Au_{12}Cu_yAg_{32-y}(SPhCl_2)_{30}$ (y = 3.74) (Co-crystallization)
Au(S1)-Au(S1)	Range: 2.770 Å---2.827 Å<br>Avg. 2.799 Å	Range: 2.752 Å---2.820 Å<br>Avg. 2.792 Å
Ag(S2)-Ag(S2)	Range: 3.090 Å---3.217 Å<br>Avg. 3.162 Å	Range: 3.074 Å---3.212 Å<br>Avg. 3.134 Å
Ag(S2)-S(S3)	Range: 2.523 Å---2.727 Å<br>Avg. 2.594 Å	Range: 2.517 Å---2.682 Å<br>Avg. 2.600 Å
M(S3)-S(S3)	Range: 2.459 Å---2.589 Å<br>Avg. 2.515 Å	Range: 2.237 Å---2.641 Å<br>Avg. 2.444 Å

Supplementary Table 3 Fitted EXAFS results of Sample 1 (slightly Au-doped M_{40} nanocluster). The data corresponds to Supplementary Fig. 29.

Sample	Path	CN	R(Å)	$\sigma^2(10^{-3}Å^2)$	ΔE_0 (eV)	R-factor
Sample 1	$Au\text{-}Au_{inner}$	3.8	2.78	8.9	4.0	0.004
	$Au\text{-}Ag_{inner}$	5.2	2.79	16.0	1.8	

Supplementary Table 4 Fitted EXAFS results of Sample 2 (h Au-doped M_{40} nanocluster). The data corresponds to Supplementary Fig. 30.

Sample	Path	CN	R(Å)	$\sigma^2(10^{-3}Å^2)$	ΔE_0 (eV)	R-factor
Sample 2	Au-S	0.8	2.27	8.1	-9.2	0.002
	$Au\text{-}Au_{inner}$	6.3	2.68	12.6	-0.8	
	$Au\text{-}Au_{outer}$	1.1	2.58	13.6	-0.8	
	$Au\text{-}Ag_{inner}$	2.7	2.76	12.6	-0.1	
	$Au\text{-}Ag_{outer}$	2.2	2.60	13.6	-0.1	

Preparation of EXAFS sample 1: 10 mg of $Ag_{28}Cu_{12}(SPhCl_2)_{24}$ was first dissolved in 10 mL of CH_2Cl_2 and then 3 mg of Au(I)-$SPhCl_2$ complexes was added in. After 1 minutes, 200 mL of hexane was poured in to pause the reaction; the precipitate was then dissolved in 5 mL of CH_2Cl_2 to yield the $Au_xAg_{28-x}Cu_{12}(SPhCl_2)_{24}$ (*Sample 1*).

Preparation of EXAFS sample 2: 10 mg of $Ag_{28}Cu_{12}(SPhCl_2)_{24}$ was first dissolved in 10 mL of CH_2Cl_2 and then 3 mg of Au(I)-$SPhCl_2$ complexes was added in. After 2 minutes, 200 mL of hexane was poured in to pause the reaction; the precipitate was then dissolved in 5 mL of CH_2Cl_2 to yield the $Au_xAg_{28-x}Cu_{12}(SPhCl_2)_{24}$ (*Sample 2*).

Crystallization of EXAFS sample 2: Single crystals of this sample were cultivated at room temperature by vapor diffusing the ethyl ether into the DMF solution of the nanocluster. After 21 days, black crystals were collected, and the structures of these nanoclusters were determined. the crystal data suggested the Au heteroatoms on *stable locations* (i.e., $Au_xAg_{28-x}Cu_{12}(SR)_{24}$, $x = 7.76$). The CCDC number of $[Au_xAg_{28-x}Cu_{12}(SPhCl_2)_{24}]^{4-}$ ($x = 7.76$) is 2083130.

Supplementary Table 5 Crystal data and structure refinement for the $[Ag_{28}Cu_{12}(SPhCl_2)_{24}]_1(PPh_4)_4$ nanocluster.

Molecular formula	$Ag_{28}Cu_{12}S_{24}C_{144}H_{72}Cl_{48}$, 4($C_{24}H_{20}P$)
Crystal system	triclinic
Space group	P -1
a/Å	21.9419(7)
b/Å	22.5717(13)
c/Å	37.2518(19)
α/°	85.543(4)
β/°	88.576(3)
γ/°	66.431(3)
Volume/$Å^3$	16858.9(15)
Z	2
ρcalcg/cm^3	1.854
μ/mm^{-1}	18.877
F(000)	9032
Radiation	CuKα (λ = 1.54186)
Index ranges	-22 ≤ h ≤ 26, -24 ≤ k ≤ 26, -44 ≤ l ≤ 27
θ range (°)	3.57 - 69.94
Measured reflections and unique reflections	204078 / 57424 (R_{int} =0.0667)
Goodness-of-fiton F^2	0.802
Largest diff. peak/hole / e $Å^{-3}$	1.2 /-1.0
Final R indexes [I>=2σ (I)]	R1 = 0.0385, wR2 = 0.0664
Final R indexes [all data]	R1 = 0.0675, wR2 = 0.0695

Supplementary Table 6 Crystal data and structure refinement for the $[Au_xAg_{28-x}Cu_{12}(SPhCl_2)_{24}]_1(PPh_4)_4$ (x = 1.32) nanocluster.

Molecular formula	$Au_{1.32}Ag_{26.68}Cu_{12}S_{24}C_{144}H_{72}Cl_{48}$, 4($C_{24}H_{20}P$), C_3H_7NO
Crystal system	triclinic
Space group	P -1
a/Å	21.800
b/Å	22.559
c/Å	37.315
α/°	85.80
β/°	88.29
γ/°	66.71
Volume/$Å^3$	16810.9
Z	2
ρcalcg/cm^3	1.871
μ/mm^{-1}	19.443
F(000)	8901
Radiation	CuKα (λ = 1.54186)
Index ranges	-24 ≤ h ≤ 26, -27 ≤ k ≤ 24, -45 ≤ l ≤ 30
θ range (°)	2.36 – 45.62
Measured reflections and unique reflections	208011 / 61054 (R_{int}=0.0440)
Goodness-of-fiton F^2	0.990
Largest diff. peak/hole / e $Å^{-3}$	6.8 / -4.1
Final R indexes [I>=2σ (I)]	R1 = 0.0444, wR2 = 0.1107
Final R indexes [all data]	R1 = 0.0612, wR2 = 0.1155

Supplementary Table 7 Crystal data and structure refinement for the $[Au_xAg_{28-x}Cu_{12}(SPhCl_2)_{24}]_1(PPh_4)_3$ (x = 7.56) nanocluster. Maybe because of the relatively poor crystal data, only three $(PPh_4)^+$ counterions were observed (although there should be four counterions in the crystal lattice due to the "-4" valence state of the $Au_xAg_{28-x}Cu_{12}(SPhCl_2)_{24}$ nanocluster).

Molecular formula	$Au_{7.56}Ag_{20.40}Cu_{12}S_{24}C_{144}H_{72}Cl_{48}$, $3(C_{24}H_{20}P)$, CH_2Cl_2
Crystal system	monoclinic
Space group	P 21/n
a/Å	21.982
b/Å	41.667
c/Å	32.188
α/°	90
β/°	102.20
γ/°	90
Volume/$Å^3$	28815.7
Z	4
ρcalcg/cm^3	2.266
μ/mm^{-1}	6.746
F(000)	18484
Radiation	MoKα (λ = 0.71073)
Index ranges	-27 ≤ h ≤ 26, -51 ≤ k ≤ 51, -39 ≤ l ≤ 25
θ range (°)	2.04 – 27.36
Measured reflections and unique reflections	141686 / 55311 (R_{int}=0.0753)
Goodness-of-fiton F^2	0.916
Largest diff. peak/hole / e $Å^{-3}$	1.4 / -1.7
Final R indexes [I>=2σ (I)]	R1 = 0.0553, wR2 = 0.1145
Final R indexes [all data]	R1 = 0.1107, wR2 = 0.1263

Supplementary Table 8 Crystal data and structure refinement for the co-crystallized nanoclusters, i.e., $[Au_4Ag_{24}Cu_{12}(SR)_{24}]_2[Au_{12}Cu_yAg_{32-y}(SPhCl_2)_{30}]_1$ (y = 3.74). Maybe because of the relatively poor crystal data, only three $(PPh_4)^+$ counterions were observed (although there should be four counterions in the crystal lattice due to the "-4" valence state of the $Au_xAg_{28-x}Cu_{12}(SPhCl_2)_{24}$ nanocluster).

Molecular formula	$2(Au_4Ag_{24}Cu_{12}S_{24}C_{144}H_{72}Cl_{48})$, $1(Au_{12}Cu_{3.74}Ag_{28.26}S_{30}C_{180}H_{90}Cl_{60})$, $10(C_{24}H_{20}P)$
Crystal system	monoclinic
Space group	P 21/c
a/Å	37.2547(5)
b/Å	41.8065(5)
c/Å	37.5795(5)
α/°	90
β/°	117.2850(10)
γ/°	90
Volume/Å^3	52017.5(12)
Z	2
ρcalcg/cm^3	1.993
μ/mm^{-1}	22.557
F(000)	29401
Radiation	CuKα (λ = 1.54186)
Index ranges	-30 ≤ h ≤ 43, -46 ≤ k ≤ 49, -44 ≤ l ≤ 22
θ range (°)	2.36 – 69.96
Measured reflections and unique reflections	197050 / 84991 (R_{int}=0.0408)
Goodness-of-fiton F^2	1.059
Largest diff. peak/hole / e Å^{-3}	7.8 / -2.7
Final R indexes [I>=2σ (I)]	R1 = 0.0681, wR2 = 0.1983
Final R indexes [all data]	R1 = 0.0926, wR2 = 0.2112

Supplementary Table 9 Crystal data and structure refinement for the $[Au_xAg_{28-x}Cu_{12}(SPhCl_2)_{24}]_1(PPh_4)_4$ (x = 7.76) nanocluster.

Molecular formula	$Au_{7.76}Ag_{20.24}Cu_{12}S_{24}C_{144}H_{72}Cl_{48}$, $4(C_{24}H_{20}P)$
Crystal system	triclinic
Space group	P -1
a/Å	21.9519(2)
b/Å	22.5476(2)
c/Å	37.2589(3)
α/°	85.4330(10)
β/°	88.4050(10)
γ/°	67.4280(10)
Volume/$Å^3$	16974.9(3)
Z	2
ρcalcg/cm^3	1.977
μ/mm^{-1}	21.376
F(000)	9529
Radiation	CuKα (λ = 1.54186)
Index ranges	-25 ≤ h ≤ 26, -26 ≤ k ≤ 26, -44 ≤ l ≤ 16
θ range (°)	5.97 – 70.37
Measured reflections and unique reflections	167072 / 59407 (R_{int}=0.0477)
Goodness-of-fiton F^2	0.982
Largest diff. peak/hole / e $Å^{-3}$	4.4 / -5.7
Final R indexes [I>=2σ (I)]	R1 = 0.0661, wR2 = 0.1650
Final R indexes [all data]	R1 = 0.0809, wR2 = 0.1738

Supplementary Table 10 Crystal data and structure refinement for the $[Au_{12}Ag_{32}(SPhCl_2)_{30}]_1[N(C_4H_9)_4]_4$ nanocluster.

Molecular formula	$Au_{12}Ag_{32}S_{30}C_{180}H_{90}Cl_{60}$, $4(C_{16}H_{36}N)$, $6(CH_2Cl_2)$
Crystal system	triclinic
Space group	P -1
a/Å	21.4072(6)
b/Å	22.7652(6)
c/Å	22.9176(6)
α/°	118.3690(10)
β/°	112.051(2)
γ/°	94.402(2)
Volume/$Å^3$	8651.3(4)
Z	1
ρcalcg/cm^3	2.425
μ/mm^{-1}	7.608
F(000)	5930
Radiation	MoKα (λ = 0.71073)
Index ranges	$-27 \le h \le 24$, $-29 \le k \le 28$, $-28 \le l \le 28$
θ range (°)	2.29 – 27.28
Measured reflections and unique reflections	106372 / 38435 (R_{int}=0.1176)
Goodness-of-fiton F^2	1.051
Largest diff. peak/hole / e $Å^{-3}$	5.4 / -5.9
Final R indexes [I>=2σ (I)]	R1 = 0.1274, wR2 = 0.2660
Final R indexes [all data]	R1 = 0.1984, wR2 = 0.3076

Supplementary Note 1 The perfect crystal data of the $[Au_{12}Cu_{y}Ag_{32-y}(SPhCl_2)_{30}]^{4-}$ nanocluster remained unavailable despite our repeated efforts, and we could only get its kernel structure while the peripheral C, H, and Cl atoms were hard to determine. Thus its crystal data and structure refinement were not provided in tables herein. Besides, the crystal date of $[Au_{12}Cu_{y}Ag_{32-y}(SPhCl_2)_{30}]^{4-}$ suggested its composition of $Ag_{12}Au_{12}Cu_{20}S_{30}$, which was untrustworthy due to the poor crystal data. Indeed, the ESI-MS data suggested its composition of $[Au_{12}Cu_{y}Ag_{32-y}(SPhCl_2)_{30}]^{4-}$ (y = 2-5; see Supplementary Fig. S13).

Supplementary References

1. Yan, J. et al. Asymmetric Synthesis of Chiral Bimetallic $[Ag_{28}Cu_{12}(SR)_{24}]^{4-}$ Nanoclusters via Ion Pairing. *J. Am. Chem. Soc.* **138**, 12751-12754 (2016).

2. Yang, H. et al. All-Thiol-Stabilized Ag_{44} and $Au_{12}Ag_{32}$ Nanoparticles with Single-Crystal Structures. *Nat. Commun.* **4**, 2422 (2013).
